# Antischistosomal Activity of Trioxaquines: *In Vivo* Efficacy and Mechanism of Action on *Schistosoma mansoni*

**DOI:** 10.1371/journal.pntd.0001474

**Published:** 2012-02-14

**Authors:** Julien Portela, Jérôme Boissier, Benjamin Gourbal, Vincent Pradines, Vincent Collière, Frédéric Coslédan, Bernard Meunier, Anne Robert

**Affiliations:** 1 Université de Perpignan Via Domitia, Perpignan, France; 2 CNRS, UMR 5244, Écologie et Évolution des Interactions, Perpignan, France; 3 Laboratoire de Chimie de Coordination du CNRS, Toulouse, France; 4 Palumed, Castanet-Tolosan, France; McGill University, Canada

## Abstract

Schistosomiasis is among the most neglected tropical diseases, since its mode of spreading tends to limit the contamination to people who are in contact with contaminated waters in endemic countries. Here we report the *in vitro* and *in vivo* anti-schistosomal activities of trioxaquines. These hybrid molecules are highly active on the larval forms of the worms and exhibit different modes of action, not only the alkylation of heme. The synergy observed with praziquantel on infected mice is in favor of the development of these trioxaquines as potential anti-schistosomal agents.

## Author Summary

Schistosomiasis is a tropical disease affecting more than 200 million people throughout the sub-tropical and tropical world. The treatment and control of schistosomiasis rely on the use of a single drug, the praziquantel and no vaccine is available. However, schistosome species with low sensitivity or resistance to praziquantel have been identified in several countries. It is an urgent need to develop new drugs against this parasite. In this context, our study reports the activity the trioxaquine PA1259. PA1259 is an hybrid drug containing two pharmacophores within a single molecule: a trioxane and an aminoquinoline. Initially developed against malaria, the trioxaquines target the heme a disposal product resulting from the digestion of the hemoglobin. The first action of the trioxaquine is an alkylation of the heme with the trioxane entity, and the second action is stacking with the heme due to the aminoquinoline moiety. In this study we show that this new drug is active *in vitro* against all schistosome stages (cercariae, schistosomule and adult). The PA1259 is also active *in vivo* and shows synergistic action in association with praziquantel. This opens the route to an efficient bitherapy of a highly neglected disease.

## Introduction

Malaria and schistosomiasis are the two most important parasitic diseases in tropical and sub-tropical areas. The parasite species responsible of these diseases are quite different: *Plasmodium* is an intracellular protozoa while *Schistosoma* is a metazoan worm. However these parasites share a common feature, they are both hematophagous. During their development in human blood stream, they digest a large quantity of host hemoglobin. As a consequence of the proteolytic digestion of this heme-containing protein, free heme is released and constitutes a major threat for both parasites due to its easy reduction by endogenous electron sources. The iron chelated by the protoporphyrin-IX ligand of heme is particularly efficient in oxygen reduction by monoelectronic transfer. This catalytic dioxygen reduction is at the origin of highly toxic reactive oxygen species (ROS). Despite their high phylogenetic divergence, convergent evolution has conducted these two parasite species to use a similar heme detoxification pathway. The hemozoin pigment, known as malaria pigment in *Plasmodium*, is an aggregation of heme dimers turning the iron inactive. Hemozoin is a dark-black inert crystalline pigment, which is structurally identical in *Plasmodium* and *Schistosoma* [1], [2].

**Figure 1:**
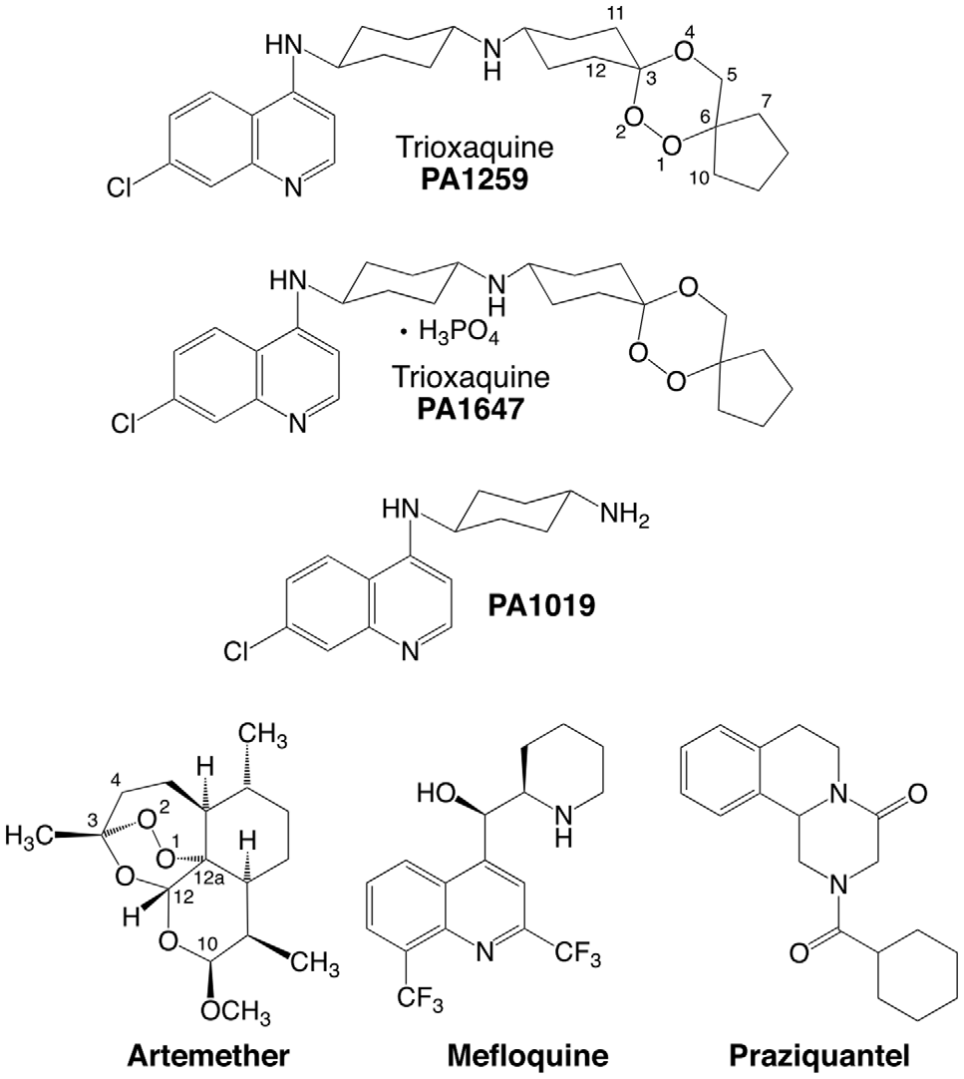
Structures of drugs. Praziquantel (PZQ), mefloquine (MFQ), artemether (ARTM), and trioxaquines PA1259 and PA1647.

The treatment and control of schistosomiasis currently rely on the use of a single drug, the praziquantel (PZQ, Figure 1). Praziquantel, a safe and effective drug, has been used for the last fourty years. However, several schistosome strains with lower sensitivity to praziquantel with possibility of resistance have been identified in African countries [3], [4]. Having a single drug to treat a disease that affects hundred millions of people is a real concern, due to the possible resistance of the parasite to this drug. As a consequence, in the last ten years important efforts have been made in either developing new drug series [5]–[7a,b], or testing existing drugs originally used on non-related diseases [8]–[13]. Among the existing medications, the antimalarial drugs targeting heme (i.e. before the hemozoin formation) are particularly interesting since free heme is not present in non-infected persons. In this field, two major series of molecules can be considered according their mechanism of action: trioxane-based molecules, that are heme-alkylating agents [14]–[17], and aminoquinoline-based molecules, that are heme-stacking agents [18], [19]. Trioxane-based molecules, whether naturally extracted or chemically synthesized, have shown moderate anti-schistosomal activities [10]–[11]. Similarly, aminoquinoline derivatives have been shown to be efficient against schistosomes experimentally infected animals, e.g. the treatment with mefloquine (MFQ) significantly reduced the number of eggs [20]. Because schistosomiasis and malaria are co-endemic in several countries, using an anti-malarial molecule against schistosomiasis might select drug-resistance in malaria parasites [8], [9]. However, many malaria patients treated with artemisinin-based combination therapy (ACT) are indeed co-infected with schistosomes. In fact, a study carried out in Côte-d'Ivoire evidenced that children infected with *S. haematobium*, treated with mefloquine-artesunate administered in accordance with the currently recommended malaria treatment schedule, showed significantly higher egg reduction rates compared to children treated with artesunate (ARTS) or mefloquine alone [21].

The future challenge in the treatment of schistosomiasis may be not to use native anti-malarial drugs but to develop new drugs considering the mechanism of action of the anti-malarial molecules targeting free heme. In these conditions, it might be useful to develop an anti-schistosomal peroxide-based drug that will be also active against malaria parasites, with the requirement that the drug should not easily induce the selection of drug-resistant strains of *Plasmodium*. This strategy should provide new molecules active on both parasites with limited side effects.

Trioxaquines (TXQ) are hybrid drugs containing two pharmacophores within a single molecule: a 1,2,4-trioxane and a 4-aminoquinoline [22]. Initially developed against malaria, they exhibit a dual mode of action: alkylation of heme with the trioxane entity, and stacking with heme due to the aminoquinoline moiety, leading to inhibition of hemozoin formation *in vitro* [23]–[25]. As reported for artemisinin derivatives [14], [15], owing to their trioxane entity, trioxaquines are indeed efficiently activated by iron(II)-heme, leading to the formation of covalent heme-drug adducts detected in the spleen of malaria infected mice [16], [24]. Because of the relationship of 1,2,4-trioxane-containing drugs with heme metabolism, we have decided to evaluate the *in vitro* activity of trioxaquines on *Schistosoma mansoni*. Several of these molecules were found highly active on both larval and mature stages of *S. mansoni* [12]. A better understanding of the molecular bases of the antischistosomal activity of trioxaquines is requested to design new active drugs, and to optimize the existing drug candidates. As a confirmation that heme is a general target of drugs active against blood-feeding parasites, we recently reported that trioxaquine PA1259 alkylates heme in female adult *S. mansoni*, and heme-drug adducts were identified from treated worms [26] (for the structure of PA1259, see Figure 1). Although important, this feature is probably not the only mode of action of trioxaquines in schistosomes. So, we decided to further evaluate their reactivity toward hemozoin, and also to attempt to have a general picture of damages induced by trioxaquines in *S. mansoni*. For this purpose, the action of a trioxaquine “prototype”, PA1259, was compared to that of three other drugs: the reference drug praziquantel (PZQ), artemether (ARTM) and mefloquine (MFQ) (Figure 1).

## Materials and Methods

### 1. Ethics Statement

The laboratory has received the permit N° A 66040 for experiments on animals from both French “Ministère de l'Agriculture et de la Pêche” and French “Ministère de l'Enseignement supérieur et de la Recherche”. Housing, breeding and animal care of the mice followed the ethical requirements of our country. The experimenter possesses the official certificate for animal experimentation delivered by both ministeries (décret n° 87–848 of October 19^th^ 1987; authorization no 007083). Animal experimentation follows the guidelines of the French CNRS. The different protocols used in this study have been validated by the French veterinary agency. Before parasite infection, mice were anaesthetized by injection of 0.1 mL/10 g of body weight of a mixture of Rompun (0.5 mL, 20 mg/mL; Bayer) and Imalgène (1.0 mL, 100 mg/mL; Rhône Mérieux) in 8.5 mL of autoclaved NaCl 8.5 (^o^/_oo_).

### 2. Parasite and host strains for antischistosomal assays

The host-parasite system used was an albino variety of *Biomphalaria glabrata* from Brazil and a strain of *Schistosoma mansoni*, also from Brazil, maintained in Swiss CD1 mice (Depré, Bourges, France). Detailed methods for mollusc and mouse infections and for parasite recovery were previously described [27].

### 3. *In vitro* antischistosomal activity

*In vitro* tests were performed on both free larval (cercariae) and parasitic stages (schistosomules and adult worms). Cercariae were recovered in spring water under binocular microscope. Parasitic stages were recovered after percutaneous infection of mice using either 120 or 400 parasite cercariae. Mice exposed to 400 cercariae were sacrificed at 21 days after infection for schistosomule recovery, while mice exposed to 120 cercariae were sacrificed at 49 days after infection for adult recovery. Schistosomules or adult worms, freshly recovered, were washed and placed in RPMI 1640 medium (supplemented with L-glutamine and Hepes 25 mM) and store in incubator chamber at 37°C.

Ten to 20 freshly recovered 21day-schistosomules or 49day-adults were placed in 24-well or in 6-well Falcon plate containing 1 mL or 3 mL of RPMI 1640 medium (supplemented with L-glutamine and Hepes 25 mM), respectively. Fifty cercariae were placed in 24-well Falcon plate containing 1 mL of spring water. The drugs PA1259, PZQ, ART or MFQ were first dissolved in DMSO to give mother solutions at 100 mg/mL. All further dilutions were done in DMSO except the last one that was realized in RPMI 1640 or spring water, for parasitic stage or free larval stage, respectively. The dilution was complemented with Tween 80 in order to obtain this final ratio dilution: culture medium/Tween80/DMSO, 1000/0.95/3.8, v/v/v. These drug solutions were added to Falcon plate that contained worms. The *S. mansoni* cultures were then incubated with each drug at final concentration of 5 or 50 µg/mL for larval stages (cercariae or schistosomules) or adult stage, respectively (a concentration of 5 µg/mL corresponds to 10, 16, 17, and 13 µM for PA1259, PZQ, ARTM, and MFQ, respectively). Control worms were treated with the same culture medium/Tween 80/DMSO, but without drug. Each test was performed in duplicate. Every 30 minutes, moving worms were counted, in order to define the percentage of survivors. Observation was extended to 8 h. Kaplan-Meier survival analyses followed by pairwise log-rank tests were used to compare survival data. Parasites showing no body contractions during a 30-s observation may be considered dead.

### 4. *In vivo* antischistosomal activity

Mice were infected percutaneously with 120 cercariae each. Twenty-one days (schistosomule stage) or 49 days (adult worm stage) post-infection, groups of five mice were treated orally. In monotherapy, PZQ or PA1259 oral treatment was performed at 100 mg/kg/d for five consecutive days, or at four doses of 50 mg/kg each, given every three hours (overall treatment period: 9 h). For bitherapy evaluation, treatments were made at the following PZQ/PA1647 wt/wt ratio: 100/0, 75/25, 50/50, 25/75, and 0/100. Administration consisted in 4 oral doses of 50 mg/kg each, given every three hours (total drug dose: 200 mg/kg). In all cases, control mice were treated with solvent but without drug. Fifteen days after treatment, mice were killed and worms were recovered by retrograde perfusion. The viscera were observed to count the worms in each mouse.

### 5. ROS production by *S. mansoni* adult worms following praziquantel or PA1259 treatment

As a preliminary, the *in vitro* concentration of PZQ or PA1259 that kills half of the parasites after one hour of incubation (LC_50_) was determined (30-secondes immobilized worms were considered as killed, since no worms recovered an activity after that 30-sec period of immobilization). For this purpose, the following ranges of concentrations were tested: 0, 0.01, 0.1, 0.5, 1, 2.5, 5 µg/mL for PZQ, and 0, 5, 10, 20, 30, 40, 50 µg/mL for PA1259. The incubation medium and drug solvents were as described above for *in vitro* treatment of schistosomes. LC_50_ values found were 50 µg/mL and 0.075 µg/mL for PA1259 and PZQ respectively. No difference was observed between male and female worms.

#### a. Hydrogen peroxide (H_2_O_2_) and superoxide anion (O_2_•^−^) quantification

Four males and two females of *S. mansoni* adult worms were disposed in a 24-well plate with 1 mL of RPMI. Worms were incubated during one hour with 0.075 µg/mL of PZQ and 50 µg/mL of PA1259 that correspond to the LC_50_ values of the drugs. Untreated worms were processed in a same manner and treated with the excipient. For each drug treatment and control experiments, ten replicate samples were analyzed. Drug treated- and untreated worms were washed 3 times with Krebs-Ringer phosphate buffer (KRPG), sonicated and the total protein amount were quantified. Fifty µL of supernatant were disposed in a 96-well plate. Hydrogen peroxide was quantified with Amplex® Red (50 µL per well, prepared according to the manufacturer's instructions). Optic density was measured with a microplate reader at 570 nm. Superoxide O_2_^.−^ was quantified with NBT (50 µl of 0.2% NBT (Sigma) dissolved in KRPG per well). The variation of OD resulting from the formation of formazan was measured with a microplate reader at 620 nm. Results were monitored at 0, 10, 20, 30, 45, 60, 90 and 120 minutes and were presented as OD values corrected by sample protein amount.

#### b. Nitric oxide (NO) quantification

Five *S. mansoni* adult females were disposed in a 24 well plate with 1 mL of RPMI and treated for 1 h with 0.075 µg/mL of PZQ and 50 µg/mL of PA1259, respectively. Worms were then washed with RPMI solution, and NO production was quantified by incubating for one hour the treated and untreated females with DAF-2DA substrate (2 µM per well). Then females were disposed on slides and observed under UV illumination using a Leica® DMLB® microscope. Pictures were taken and analyzed by using the imageJ software [28]. Briefly DAF-2 fluorescence intensity was quantified over the entire worm surface and results were expressed as mean DAF-2 fluorescent intensity/pixel. For each female picture, mean DAF-2 fluorescent intensity was calculated by separating the Red image from the Green, and Blue ones (RGB). Indeed Red-image was more appropriated to calculate fluorescence since DAF-2 fluorescent background was more attenuated.

### 6. Chemicals

PA1259 and PA1019, synthetized as reported in the patent application WO/2007/144487, and PA1647 were provided by Palumed. PA1647 is the diphosphate salt of PA1259 (stoichiometry checked by CHN-elemental analysis). Artemether (ARTM) was a gift from Rhône-Poulenc Rohrer Doma (Antony, France). Praziquantel (PZQ) and mefloquine (MFQ) were purchased from Sigma-Aldrich, as well as sodium dithionite (Na_2_S_2_O_4_), dimethylsulfoxyde (DMSO, ACS spectrophotometric grade ≥99.9%), pyridine (≥99%), and methanol (Chromasolv ≥99.9%). Formic acid (99+%) was from ACROS. Hemin (ferriprotoporphyrin IX chloride, 98.0%) from bovine blood was purchased from Fluka (Switzerland). All chemicals and solvents were used as purchased without further purification. Milli-Q water (resistivity ≥18.2 MΩ) was used for LC-MS eluent preparation.

### 7. Extraction of heme and heme-drug adducts from *S. mansoni* worms

Adult *S. mansoni* females were recovered in mice seven weeks after infection, and maintained in culture in RPMI 1640 medium, supplemented with L-glutamine and Hepes 25 mM, at 37°C. Groups of twenty-five schistosomes, freshly recovered, were carefully washed with RPMI 1640 medium, then treated with PA1259 or ARTM at 50 µg/mL for 3 hours. Then, worms were crushed with sand, and the obtained powder was extracted with pyridine (500 µL). The mixture was vigorously stirred (vortex), placed in an ultrasonic bath for 30 min and, finally, magnetically stirred at 37°C overnight. After centrifugation at 4000 rpm for 30 min, the supernatant was withdrawn and filtered through ptfe 0.45 µm syringe filters before analysis by HPLC or LC-MS.

### 8. LC-MS analysis

#### a. HPLC conditions for heme-drug adducts

The LC-MS analyses were performed using a Agilent 6140 equipment (HPLC column: 5 µm C18 X-Bridge, 150×4.6 mm, Waters; linear elution gradient from water/formic acid, 100/1, v/v to methanol/formic acid, 100/1, v/v in 30 min; flow rate = 1 mL×min^−1^; injection volume: 100 µL; UV-vis. detection at 398 nm (*λ*_max_ of heme), and ESI^+^-MS detection). The analytic conditions were previously optimized by using chemically prepared heme-PA1259 adducts [3.2 mg of Fe^III^(PPIX)Cl, 10 molar equivalent of sodium dithionite and 1.5 molar equivalent of trioxaquine PA1259 were dissolved in 500 µL DMSO. The reaction was carried out at 37°C, under an argon atmosphere, for 2 h].

#### b. Mass spectrometry conditions

The ionization was performed in electrospray mode, using an Agilent 6410 triple quadrupole setup (source temperature  = 350°C, gas flow nebulizer  = 13 L.min^−1^). The detection and quantification were performed in scanning mode (total ionic current) with a step size of 0.1 atomic mass units (amu) and scan range of 300–1200 amu.

### 9. Electronic Microscopy

#### a. Scanning electron microscopy (SEM)

Schistosomes were fixed with 2% glutaraldehyde in Sorensen buffer (0.1 M, pH = 7.4) for 1 hour, washed with cacodylate buffer (0.1 M sodium cacodylate) for 12 hours. After dehydration and critical point drying, they were mounted on microscope stubs, followed by platinum sputtering during 160 seconds. We used a SEM-FEG microscope (JEOL JSM 6700F) with an accelerating voltage of 30 kV. The head of schistosomes was first located, and photographs were then recorded, with magnification ×1000, ×2000, or ×5000 (50 photographs per worm; two worms for each treatment).

#### b. Transmission electron microscopy (TEM)

Schistosomes were fixed with 2% glutaraldehyde in Sorensen buffer (0.1 M, pH = 7.4) for 1 hour, washed with the same buffer for 12 hours. They were postfixed with 1% OsO_4_ in Sorensen buffer 0.05 M and saccharose 0.25 M for 1 hour. Schistosomes were dehydrated by incubation in successive aqueous ethanol solutions containing an increasing proportion of ethanol up to 100 vol%, and then embedded in LR Whyte resin (medium grade acrylic resin, Electron Microscopy Sciences). After 48 h of polymerization at −20°C under UV irradiation, ultrathin sections (50 nm) were mounted on 150 mesh collodion-coated copper grids and poststained with 3% uranyl acetate in 50% ethanol, and with 8.5% lead citrate, before being examined on a HU12A Hitachi electron microscope, at an accelerating voltage of 75 kV.

#### c. X-ray analysis

The presence of iron in hemozoin pellets was assessed by using a TEM-FEG (JEOL JEM 2100F) electronic microscope, with an accelerating voltage of 200 kV, equipped with an element analyzer (PGT, resolution 136 eV). This analysis has been performed on the ultrathin sections of schistosomes previously analyzed by TEM.

## Results

### 1. *In vitro* activity of trioxaquine PA1259 on free and parasitic stages of *S. mansoni*

**Figure 2:**
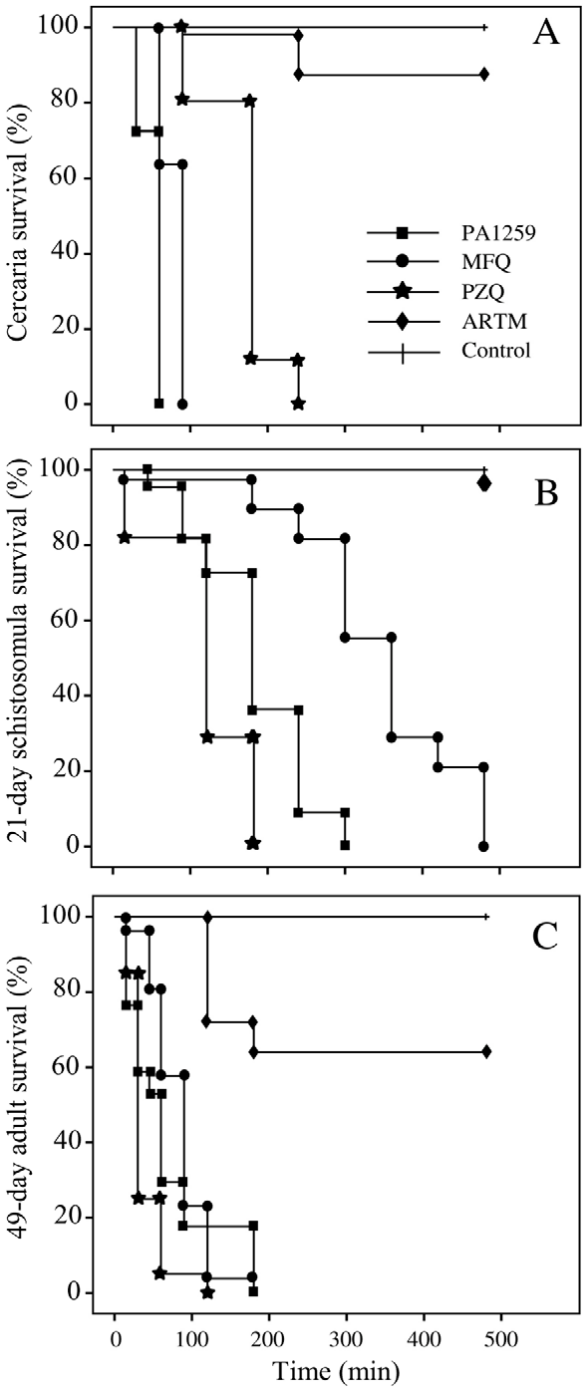
Comparative *In vitro* activities. PA1259 (▪), praziquantel (★), artemether (⧫), and mefloquine (•) on cercariae (A), on 21-days old schistosomules (B) and on 49-days old adult (C) *S. mansoni*. Cultured larvae (cercariae or schistosomules) or adults were treated with compounds at 5 µg/ml or 50 µg/ml, respectively.

The *in vitro* activities of PA1259 (▪), praziquantel (★), artemether (⧫), and mefloquine (•) on cercaria-, 21 day-old schistosomules, and 49 day-old adult worms are reported in Figure 2A, 2B, and 2C, respectively, as Kaplan-Meier plots. When treated with PZQ at 5 µg/mL, after 4 h of contact with the drug, all cercaria were immobilized for a 30 s-observation time. To obtain such immobility after treatment with PA1259 or MFQ required only 60 or 90 min, respectively. In the presence of ARTM, 8 h after treatment, more than 80% of cercariae were still moving (Figure 2A). The treatment of 21-day schistosomules with PZQ at 5 µg/mL resulted in a complete immobilization of all larvae after 3 h of contact. With PA1259 or MFQ, the same effect was obtained after 5 h and 8 h, respectively. When ARTM was used in the same conditions, no significant effect was observed after 8 h (Figure 2B). The treatment of 49-day adult schistosomes with drugs at 50 µg/mL resulted in complete immobilization of all worms in 2 h in the presence of PZQ, and 3 h in the presence of MFQ or PA1259. With ARTM at the same concentration, more than 60% of worms were still moving after 8 h (Figure 2C).

In addition, the activity of PA1019, which is the 4-aminoquinoline residue contained in PA1259, was evaluated on schistosomules and adult worms. Seven hours of contact of PA1019 was required to immobilize only 27% of schistosomules and 79% of adult worms.

### 2. Activity of trioxaquine PA1259 on mice infected by *S. mansoni*

In mice infected by *S. mansoni*, the reduction of worm burden upon oral treatment by PA1259 or PZQ is reported in Table 1. In mice treated with PA1259 at five daily doses of 100 mg/kg starting from day 21 post-infection, 26.6±5.4 worms were collected 15 days later. When treatment was done at day 49 post-infection, 21.0±7.0 worms were collected. These values correspond to a reduction of 31% and 42% on larval and adult stage, respectively, with respect to mice treated by excipient alone (control mice). With the same treatment schedule, PZQ induced a worm burden reduction of 20% and 79% on schistosomules and adult worms, respectively. With four doses of 50 mg/kg of PA1259 given every three hours, the reduction of the worm burden was 53% (16.8±7.2 worms) or 40% (20.0±5.1 worms) on larval stage or adult stages, respectively. The same treatment schedule but with PZQ induced a worm burden reduction of 41% and 86% on schistosomules and adult worms, respectively. These treatments did not induce any visible adverse effect in mice.

**Table 1:** *In vivo* effect of PA1259 and praziquantel.

Treatment	Administration	Schistosomula		Adult	
		Mean number of worms ± SD	Worm reduction	Mean number of worms ± SD	Worm reduction
Control		38.6±5.4	_	33.4±5.0	_
PA1259	5×100 mg/kg^a^	26.6±5.3	31%*	21.0±7.0	42%*
PZQ	5×100 mg/kg^a^	30.7±2.4	20%	7.4±7.8	79%*
Control		35.4±5.0	_	33.4±5.0	_
PA1259	4×50 mg/kg^b^	16.8±7.2	53%*	20.0±5.1	40%*
PZQ	4×50 mg/kg^b^	21.0±6.0	41%*	4.8±6.5	86%*

Effect on worm recovery of two administration protocols of PA1259 and praziquantel (PZQ) administered to mice harbouring either a 21-day-old schistosomula or 49-day-old adult *S. mansoni* infection.

a The five doses of 100 mg/kg were administered daily during five consecutive days.

b The four doses of 50 mg/kg were administered every three hours (overall treatment period: 9 h).

*Significant difference compared to the control group at 5% level (Mann-Whitney U test). SD stands for standard deviation.

### 3. Trioxaquine - praziquantel association on mice infected by *S. mansoni* schistosomules

The effect of a combination of PZQ and trioxaquine PA1647 on the reduction of the worm burden in *S. mansoni* infected mice is reported in Table 2. The first course consisted of 4 oral treatments with 50 mg/kg of drug given every three hours. The drug combinations were 100% of PZQ (line 2), 75 wt% PZQ/25 wt% PA1647 (line 3), 50 wt% PZQ/50 wt% PA1647 (line 4), 25 wt% PZQ/75 wt% PA1647 (line 5), and 100% of PA1647 (line 6), respectively. In these conditions, the reduction of the schistosomules burden with respect to control mice was 24% with 100% PZQ (line 2), 73% with 75 wt% PZQ/25 wt% PA1647, (line 4), and 18% with 100% of PA1647 (lines 2, 4, and 6, respectively). These treatments did not induce any visible adverse effect in mice.

**Table 2:** *In vivo* effect of PA1647 combined with praziquantel on 21-day-old schistosomula.

		21-day Schistosomula	
	Treatment	Mean number of worms ± SD	Worm reduction
1	Control (excipient alone)	43.4±4.9	_
2	4× (PZQ 50 mg/kg)	33.0±1.6	24%
3	4× (PZQ 37.5 mg/kg + PA1647 12.5 mg/kg)	31.0±2.7	29%
4	4× (PZQ 25 mg/kg + PA1647 25 mg/kg)	11.6±3.9	73%*
5	4× (PZQ 12.5 mg/kg + PA1647 37.5 mg/kg)	31.5±3.1	27%
6	4× (PA1647 50 mg/kg)	35.8±4.4	18%

The treatment schedule consisted in four doses of 50 mg/kg (sum of the two drugs) every three hours.

*Significant difference compared to the control group at 5% level (Mann-Whitney U test). SD stands for standard deviation.

### 4. Microscopy

Adult *S. mansoni* females were treated in *vitro* with PA1259, PZQ, ARTM or MFQ at 50 µg/mL for three hours, and compared with untreated worms (excipient only).

#### a. Photon microscopy

**Figure 3:**
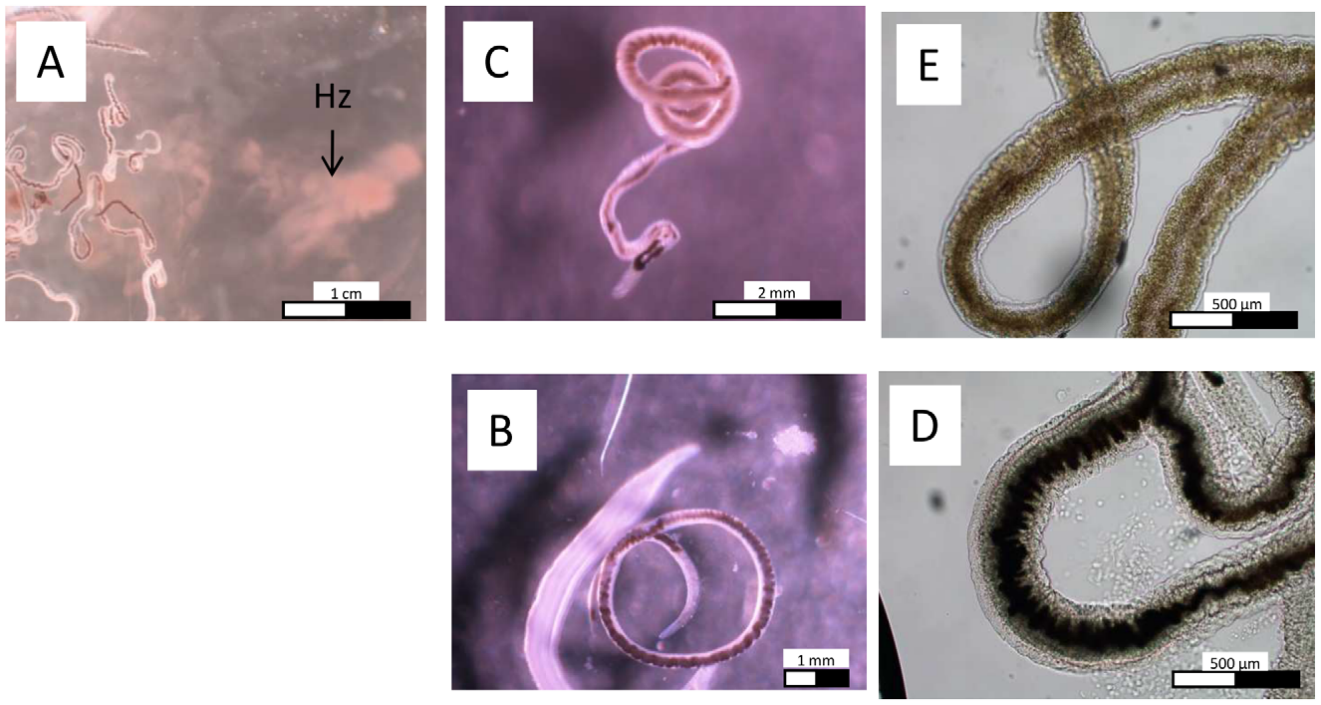
Optic microscopy of treated worms. *S. mansoni* females treated with PA1259 at 50 µg/mL (A, C, and E), compared to control worms (B and D). Hz stands for hemozoin. In B and D: black Hz in a well defined gut; in C: Few minutes after treatment, Hz turns to brown inside the gut; in E) one hour after treatment the brown pigment was transfered outside of the gut.

Upon treatment with trioxaquine PA1259 at 50 µg/mL, a brown cloud of small dark crystals readily appeared in the culture medium (right part of the Figure 3A). Few minutes after treatment, the black color inside the gut of worms (Figure 3D and 3E, untreated worms) turned to light brown (Figure 3B). After one hour, the gut walls of treated worms were no more visible, and the brown pigment invaded the whole worm body (Figure 3C). Such a modification was observed with none of the reference drugs, namely PZQ, ARTM, or MFQ.

#### b. Scanning electron microscopy (SEM)

First of all, the general morphology of worms was rather affected, especially after treatment with mefloquine or trioxaquine PA1259 (Figure S1). Worms treated with PA1259 were convoluted (Figures 3C and S1c). The schistosomes treated by mefloquine seemed stiff, contracted (with a mean diameter significantly reduced with respect to untreated, Figure S1d). Worms treated with PZQ exhibited local drastic swelling (encircled zone in Figure S1b). In some cases, the breaking of the worm occurred during the preparation for microscopy. These events, not related to the drug treatment, are indicated as white crosses in Figure S1.

Second, chistosomes exhibit spines along all the length of body, but with a higher density in the hindbody and forebody parts with respect to midbody part, and on the dorsal side with respect to the ventral one (Figure S7). The ciliated sensory papillae were also well visible. The treatment with trioxaquine PA1259 induced a drastic disorganization of spines, which became rough and bushy (Figure S2c). In some places, the disappearance of spines, along with dilated spine sockets, was observed, suggesting that spines have been drawn back (Figure S3c). The spines were nearly unchanged upon treatment with PZQ or ARTM (Figure S2, panels b and e, respectively). However, PZQ induced damages to sensory papillae that became subsided, losing their domed shape and their sensory cilium (arrows in Figure S2b and Figure S3b). Extensive blistering occurred with ARTM (arrows in Figure S3e). Treatment with MFQ induced a cavernous aspect of the worm, with disappearance of spines, and complete destruction of the external structures (Figure S2d).

**Figure 4:**
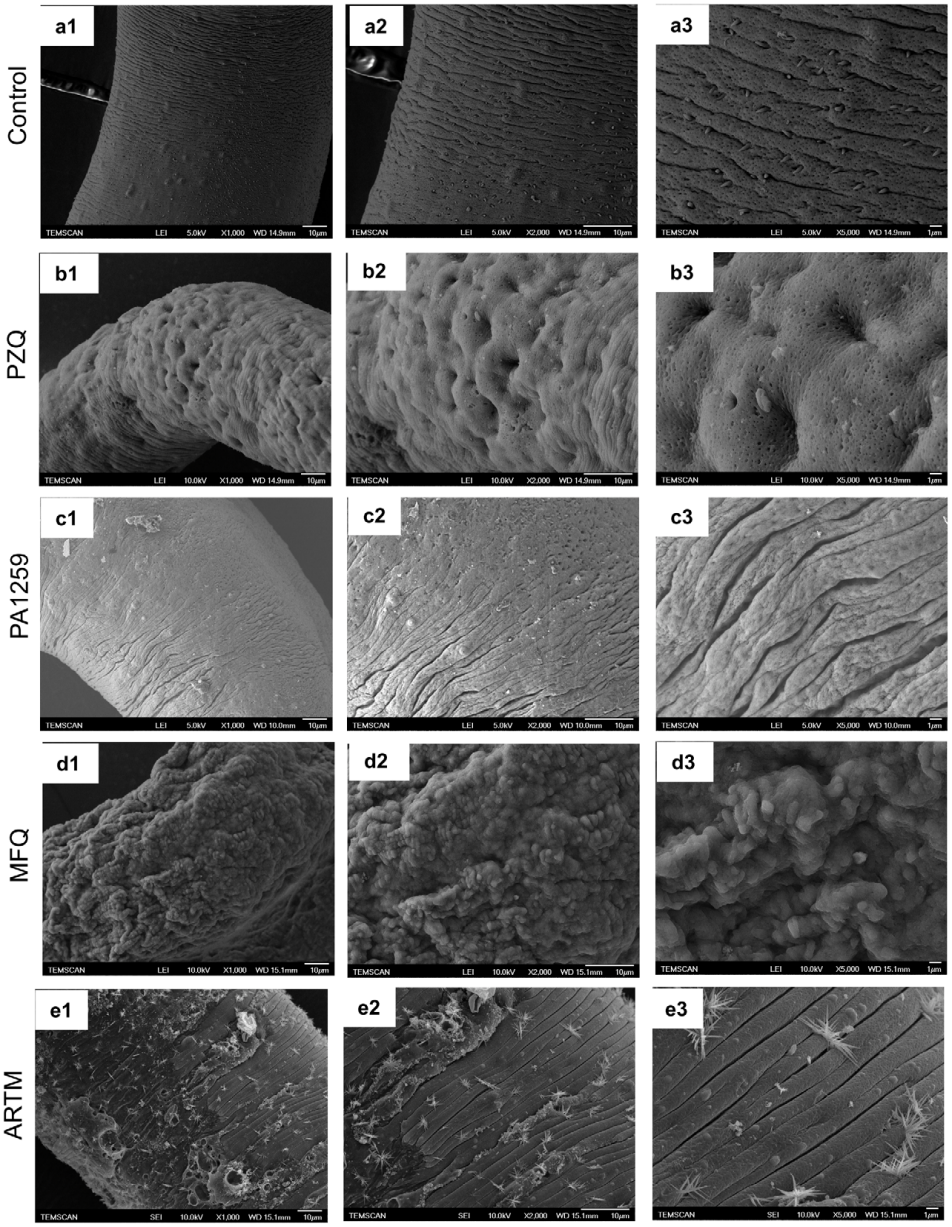
SEM images of the mid-body region of *S. mansoni* adult females. Control worms (a), compared to worms treated with b) praziquantel (PZQ), c) trioxaquine PA1259, d) mefloquine (MFQ), or e) artemether (ARTM). Magnification ×1000 (a1-e1), ×2000 (a2-e2), ×5000 (a3-e3); the bars stand for 10 µm.

Third, the tegument of control schistosomes exhibited normal circular ridges with regular clefts (Figure 4a). After treatment with PZQ or trioxaquine PA1259, the transverse tegumental ridges encircling the body worm were not observable any longer. Swelling induced loosing of the clefts, and fusions of ridges were always observed (Figure 4, panels b and c, respectively, and S4). Ridges were damaged, but still visible after treatment by artemether (Figure 4e and S4e), in contrast to females treated with praziquantel or PA1259, which showed a complete fusion of the ridges. PZQ mainly induced swelling and formation of holes, probably corresponding to damaged sensory papillae (arrow in Figure S3b). In the case of PA1259, the fusion of ridges was accompanied by formation of long irregular and disorganized splits (arrows in Figure S4c). Worms treated with PA1259 were also characterized by swelling, and dilated and/or empty spine sockets were visible (Figure S3c). After treatment with artemether, there were both rather undamaged regions, with well-conserved ridges and regions showing extensive vesiculations, burst of blebs, erosion of the tegument and sloughing (Figures 4e and S3e). The extensive blistering observed upon treatment with artemether was not present with the other drugs. Blebs were so large that they fused together, inducing a detachment of large parts of the tegument layer, and splits parallel to the ridges. Magnification of this peeling phenomenon is depicted in Figure S5b, and compared with erosion and peeling subsequent to PA1259 treatment (Figure S5a). In the latter case, focal sloughing was also present, but no rest of the tegumental layer could be observed. After ARTM treatment, round holes, about 1 µm large, were also visible in the underlying layer (Figure S5b). MFQ treatment induced the most destructive damages with a cavernous aspect of worm surfaces (Figures 4d and S4d). In addition, whether the treatment, the oral and ventral suckers were affected in most of the worms examined, showing serious deformations and extensive swelling (data not shown).

#### c. Transmission electron microscopy of S. mansoni (TEM)

By ultramicrotomy, ultrathin (50 nm) transverse sections of *S. mansoni* adult females were prepared. For each drug treatment, three worms were analyzed by TEM (four sections prepared for each worm).

**Figure 5:**
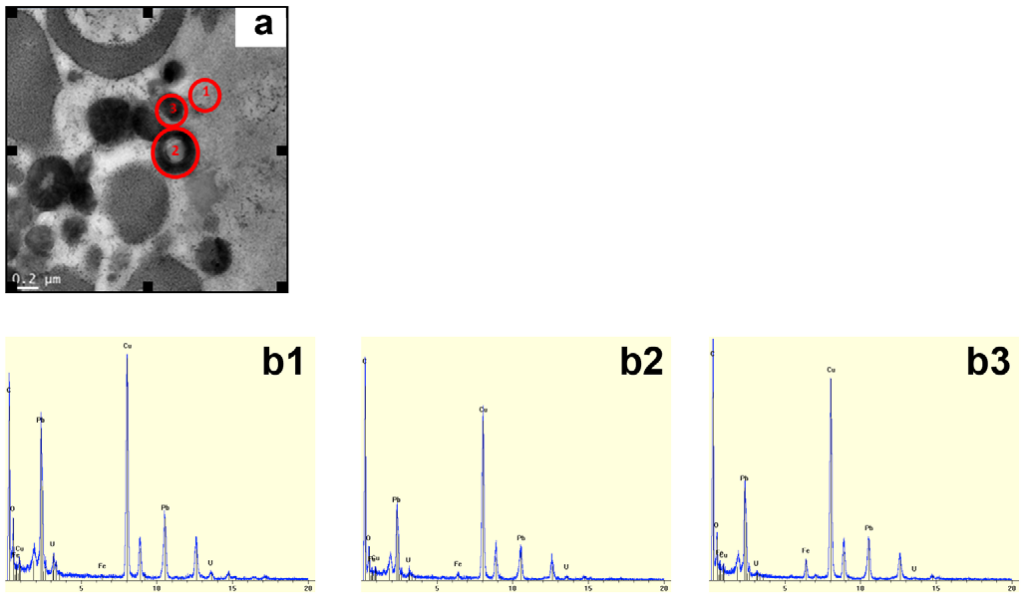
Hemozoin detection. (a) TEM image of hemozoin pellets of untreated *S. mansoni* female. (b) Element analysis of 3 differents part of the picture (a): b1. Background: low contrasted zone, b2. Hemozoin pellets in early stage formation, b3. Hemozoin pellets at final stage.

First, the hemozoin pellets appeared as 200–300 nm large contrasted dark grey disks. Early crystallization of hemozoin was visualized at the surface of lipid droplets giving dark grey circles with light grey inside [29]. The presence of iron was assessed by energy dispersive (X-ray) microscopy in mature- or in formation-hemozoin pellets (noted 3 and 2, respectively, in Figure 5a). By contrast, no iron was detected in many uncontrasted locations (noted 1 in Figure 5a).

**Figure 6:**
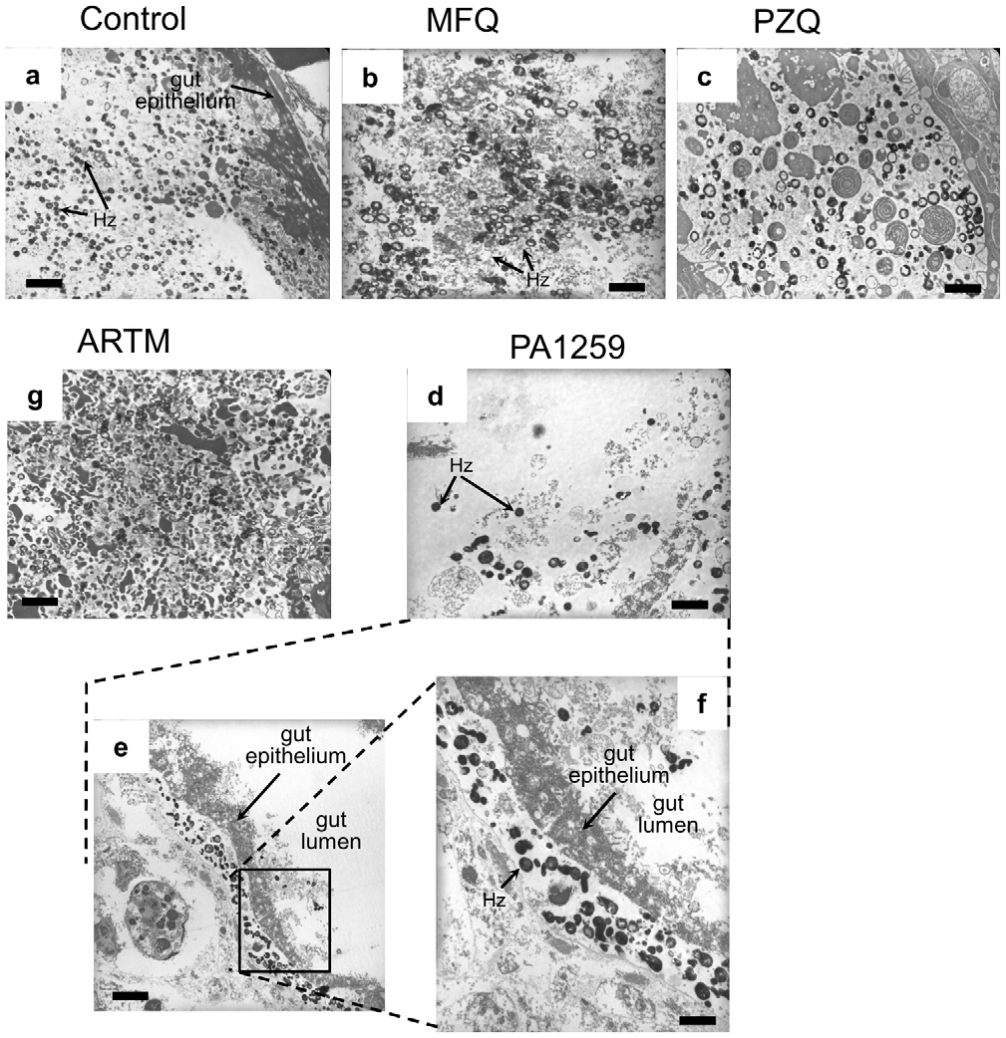
TEM images of hemozoin (Hz) inside the gut. Control worms (a), compared to worms treated with b) mefloquine (MFQ), c) praziquantel (PZQ) or d, e, f) trioxaquine PA1259, and g) artemether. The scale bars stand for 2 µm in panels a, b, c, d, f, and g, and for 5 µm in panel e.

Images obtained for control worms, compared with those obtained after treatment with mefloquine (MFQ), praziquantel (PZQ), and trioxaquine PA1259 are depicted in Figure 6. The gut of *S. mansoni* adult females treated with PA1259 (panel d) exhibited a drastically lower hemozoin content with respect to control worms (panel a). Upon treatment with MFQ or PZQ, the hemozoin content was significantly reduced with respect to control worms (panels b and c, respectively), but in much lower proportion than upon treatment with PA1259. In addition, upon treatment by PA1259, a part of remaining hemozoin pellets migrated across the epithelium and was found lining its external membrane (Figure 6, panels e and f).

Second, the vitelline cells of control female worms were well characterized (Figure S6a). Ultrastructure of vitelline cells of worms treated with PZQ and ARTM exhibited focal fusion of vitelline balls in vitelline droplets (vd on Figure S6). However, the most part of vitelline cells remained intact. By contrast, extensive alterations appeared in schistosomes treated with PA1259 and, in a greater extent with MFQ (panels c and d, respectively). Fusion in the process of vitelline balls (arrows on Figure S6, panel c1), and formation of lipid droplets were seen (ld in panel d2).

**Figure 7:**
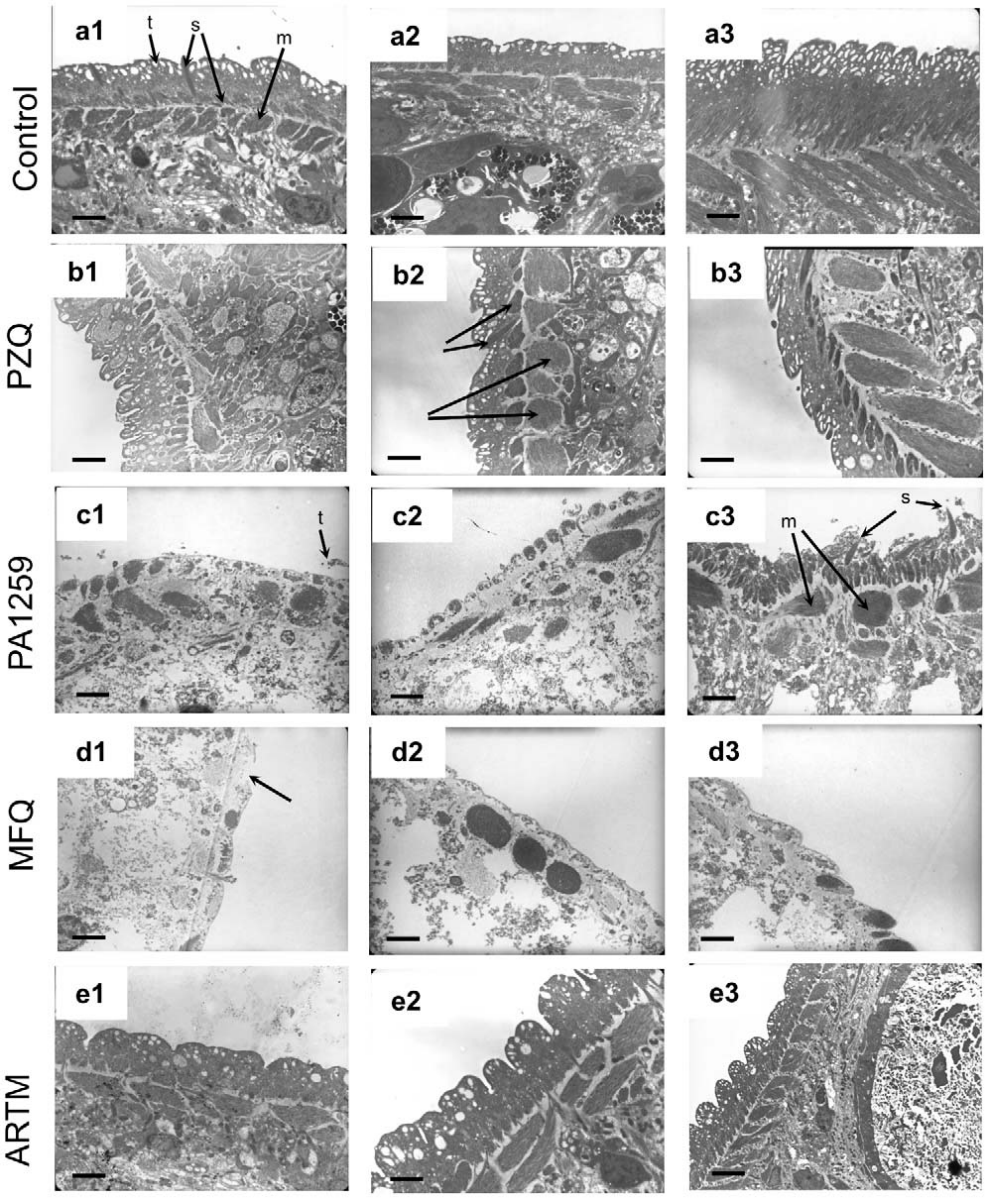
TEM images of tegument. Control worms (a), compared to worms treated with b) praziquantel (PZQ), c) trioxaquine PA1259, d) mefloquine (MFQ), or e) artemether (ARTM). The scale bars stand for 2 µm in panels a1-e1, and for 1 µm in panels a2-e2.

Third, the tegumental matrix (t), spine and spine sockets (s), and muscle bundles (m) were clearly visible on many transmission electron micrographs of control worms (Figure 7, panels a). After treatment with PZQ, the extent of tegumental damages was significantly different depending on the parasite sections. However, in the most part of cases, the tegumental matrix was slightly modified, but significant swelling of the underlying muscle bundles occurred (Figure 7, panels b). Treatment with PA1259 (Figure 7, panels c) caused complete disappearance of the tegument matrix and severe swelling and disorganization of the sub-tegumental muscle layer (m). Some spines were still visible (s). The severity of tegumental damages induced by treatment with MFQ are visible in Figure 7d. All the worm sections exhibited complete lysis of tegument, extensive disappearance of spines, and collapse of internal structures. ARTM induced deep clefts in the tegument, but the damages were drastically less important than those caused by PA1259 or MFQ (Figure 7, panels e).

### 5. Molecular mechanism of damages induced by antischistosomal drugs

#### a. Reduced or radical oxygen species: NO•, O_2_•^−^, H_2_O_2_

**Figure 8:**
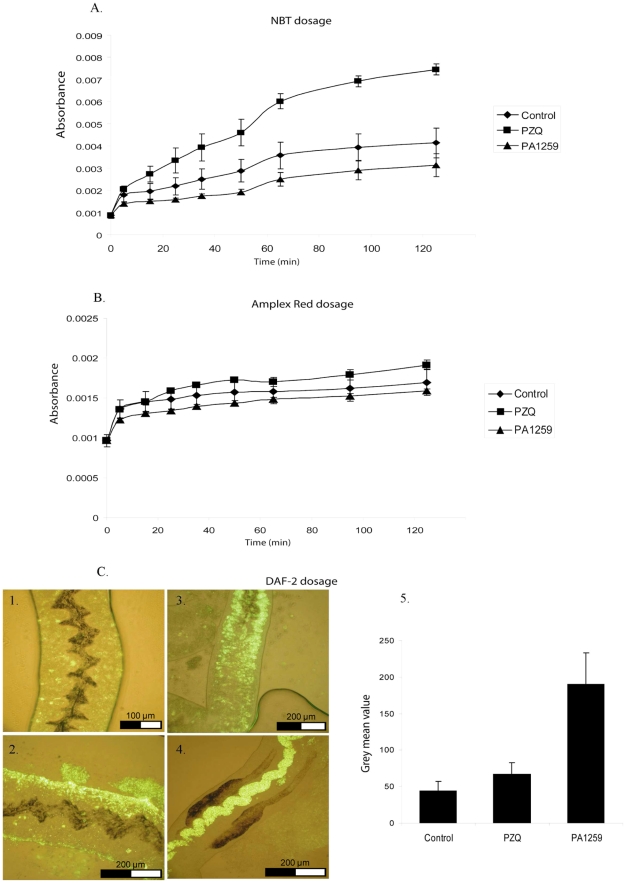
Reduced or radicals oxygen species dosage. A. Superoxide anion (O_2_•^−^) was dosed using Nitro Blue Tetrazolium method B. Hydrogen peroxide (H_2_O_2_) was dosed using Amplex Red method. B. Nitric Oxide (NO•) was dosed using DAF-2 method.

Dosage of reduced oxygen species O_2_•^−^, H_2_O_2_, and NO•, is reported in pannels A, B, and C of Figure 8, respectively. In worms treated with PZQ, the production of O_2_•^−^ was roughly twice higher than that of control worms (Figure 8A: PZQ (▪): OD = 0.0075, Control (⧫): 0.0041). By contrast, treatment with PA1259 (▴, OD = 0.0031) did not improve the production of O_2_•^−^ with respect to control. The production of H_2_O_2_ whether control, PZQ, or PA1259 treated worm, was not significantly different (optic density values being 0.0017, 0.0019, and 0.0016 for control (⧫), PZQ (▪), and PA1259 (▴)-treated worms, respectively; Figure 8B). The production of NO• was very low in control worms (Figure 8, panel C1, intensity 44±14, pannel C4). Females treated with PZQ exhibited a slightly enhanced fluorescence due to NO• located near the tegument (Figure 8, panel C2, intensity 67±16, pannel C4), whereas, in females treated with PA1259, the fluorescence due to NO• was very high, and mainly detected inside the gut and near the vitelline cells (Figure 8, panel C4 and C3, respectively, intensity 190±43, panel C5).

#### b. Characterization of covalent heme-drug adducts

**Figure 9:**
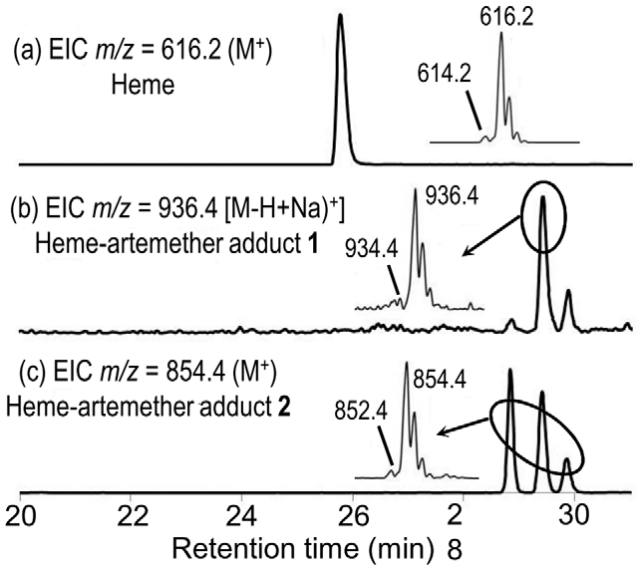
LC-MS analysis of the extract of *Schistosoma mansoni* treated with artemether (50 µg/mL). Extracted ionic current (EIC) traces for: (a) *m/z* = 616.2 (Heme, M^+^); (b) *m/z* = 936.4 (“Complete” heme-artemether adduct **1**, (M-H+Na)^+^; (c) *m/z* = 854.4 (heme-artemether adduct **2**, MH^+^). (Inserts correspond to the mass spectra of circled chromatographic peaks).

The LC-MS profile of the extract of worms treated by ARTM is depicted in Figure 9. Chromatographic peaks with retention times 28.8, 29.4, and 29.9 min exhibited an intense ionic current with *m/z* = 854.4 amu (*z* = 1) (compound **2**, Figure 9c). Trace amounts of a compound having *m/z* = 936.4 amu was also detected (compound **1**, Figure 9b).

**Figure 10:**
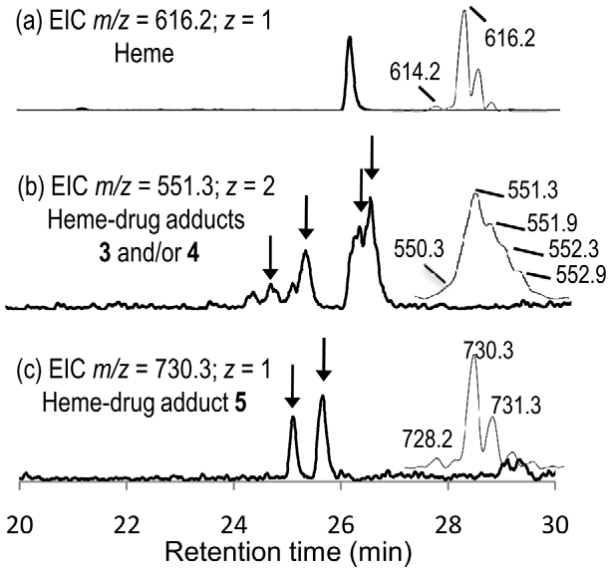
LC-MS analysis of the extract of *Schistosoma mansoni* treated with trioxaquine PA1259 (50 µg/mL). Extracted ionic current (EIC) traces for: (a) *m/z* = 616.2 (Heme, M^+^); (b) *m/z* = 551.3 (“Complete” heme-PA1259 adducts **3** and/or **4**, MH^+^/2); (c) *m/z* = 730.3 (heme-PA1259 adduct **5**, MH^+^). (Inserts correspond to the mass spectra of chromatographic peaks with arrows).

The LC-MS analyses of extracts of *S. mansoni* worms treated with PA1259 are reported in Figure 10a-c. Along with the ionic current of heme [R_t_ = 26.1 min, *m/z* = 616.2, *z* = 1, M^+^ for Fe^III^(PPIX), Figure 10a], several chromatographic peaks were detected at R_t_ = 24.7, 25.3, 26.3, and 26.5 min, with *m/z* = 551.3 and *z* = 2, having an exact mass value of 1101.2 amu (Compounds **3**, **4**, Figure 10b). Another product **5** was also detected (R_t_ = 25.1 and 25.7 min, *m/z* = 730.3, *z* = 1, Figure 10c).

#### c. Heme oxidative cleavage in S. mansoni

Twenty five freshly recovered *S. mansoni* adults were maintained in RPMI for 3 h, but without drug treatment, and then extracted with pyridine as described above. The LC-MS analysis of the extract exhibited the presence of two chromatographic peaks with the same *m/z* value of 347.1 amu (R_t_ = 13.8 and 14.1 min). We failed to detect a significant amount of these compounds in schistosomes treated with ARTM or MFQ under similar conditions.

## Discussion

### 1. *In vitro* activity of trioxaquine PA1259 on free and parasitic stages of *S. mansoni*

The *in vitro* activities of PA1259 (▪) on cercariae, schistosomules, and adult worms *S. mansoni* are reported in Figure 2A, 2B, and 2C, respectively, along with activities of praziquantel (★), artemether (⧫), and mefloquine (•) given as comparison. Results depicted in Figure 2 show that PA1259 exhibits a significant anti-schistosomal activity on all parasite stages. Concerning the free cercarial stages, PA1259 and MFQ have similar efficacy (all cercariae were dead within 60–90 min, Figure 2A). These two drugs are significantly more efficacious than PZQ (after 90 min of treatment with PZQ, 80% of cercariae were still moving, and 4 hours was needed to immobilize all cercariae). On the schistosomules stage, the time required to kill all schistosomules was 3 h, 5 h, or 8 h, with PZQ, PA1259, or MFQ, respectively (Figure 2B). So, PZQ and PA1259 are more active than MFQ. On adult parasites, the activities of these three last molecules are not significantly different (p>0.05), with all worms killed at 2–3 h (Figure 2C). The *in vitro* activities obtained for both MFQ and PZQ are consistent with previous reports [30], [31]. Compared to MFQ, PA1259 is more potent on schistosomule stage, and has the same activity on adult stage. It is noteworthy that MFQ, based on a quinoline moiety, is active on the cercarial stage, whereas ARTM, containing a trioxane, is not. This feature suggests that the quinoline part of PA1259 may play a role in its activity against schistosomes (especially the cercarial stage), and that the parasite heme is not the only target of this drug (cercariae do not contain heme). In fact, a non-heme target has recently been proposed for MFQ [30]. ARTM is inactive on cercariae and schistosomules, and only poorly active on adult worms. In fact, this latter drug was reported to be active only when hemin was added in the culture medium [32].

In addition, the activity of PA1259 on schistosomules and adult worms was compared with that of PA1019 which is the 4-aminoquinoline residue contained in PA1259 (see Figure 1 for the structure of PA1019). PA1019 was only poorly active, and 7 h were required to immobilize only 27% of schistosomules and 79% of adult worms. By contrast, treatment with PA1259 immobilized 100% of schistosomules and adult worms after 5 h or 3 h, respectively. This result support an additive or synergistic effect of the quinoline and trioxane moieties of PA1259.

### 2. Activity of trioxaquine PA1259 on mice infected by *S. mansoni*

In *S. mansoni* mice, the reduction of worm burden upon oral treatment by PA1259 or PZQ is reported in Table 1. The administration of trioxaquine PA1259 at five daily doses of 100 mg/kg resulted in a reduction of 31% and 42% on larval and adult stage, respectively. With four doses of 50 mg/kg every three hours, the reduction of worm burden was 53% or 40% on larval or adult stages, respectively. Whatever the used protocol, the activity of PA1259 on larval stage was very close (slightly higher) to that of PZQ. On adult stage, PA1259 exhibited a significant activity, with a reduction of the worm burden being half of that obtained with PZQ. It is noteworthy that the efficacy of PA1259 was very close on schistosomules and adult worms, whereas PZQ exhibited a significantly higher efficacy on adult schistosomes compared to schistosomules. This feature suggests a different mode of action for these two drugs. For comparison purpose, quinoline- or artemisinin-based molecules present variable efficacy. For instance, among quinoline-based molecules, MFQ exhibits a significant efficacy against schistosome infections, but chloroquine is inactive against schistosome infections [8]. For comparison, artemisinin derivatives or synthethic trioxolanes mainly possess activity against schistosomules [33], [34]. In a mouse model, ARTM was reported to be inactive at 800 mg/kg on adult *S. mansoni* infection [33].

In addition, on *S. mansoni* schistosomules, 4 doses of 50 mg/kg of PA1259 every three hours (total dose of 200 mg/kg given over a period 9 hours) was found to be a more efficacious protocol than a total dose of 500 mg/kg given over a period of 5 days (100 mg/kg administered daily).

### 3. Trioxaquine - praziquantel association on mice infected by *S. mansoni* schistosomules

Owing to the complementarity of PZQ and trioxane based drugs against schistosomes, we investigated the reduction of the worm burden in mice infected by 21-day *S. mansoni* schistosomules when orally treated with an association of PZQ and trioxaquine PA1647, the diphosphate salt of PA1259 (Figure 1). Five courses were done with different proportions of PZQ and PA1647. Each course consisted of four oral doses of (PZQ + PA1647), at a total amount of 50 mg/kg for each dose (Table 2). The reduction of the schistosomule burden with respect to control mice was 73% with 50 wt% PZQ/50 wt% PA1647, (corresponding to 32 mol% of PA1647, line 4). For comparison, it was only 24% or 18% when PZQ or PA1647, respectively, were used as monotherapy (lines 2 and 6, respectively). A simple additive effect between PZQ (24% worm burden reduction at 50 mg/kg), and PA1647 (18% worm burden reduction at 50 mg/kg) would have provided at best a (24+18)/2 = 21% worm reduction with the combination PZQ 25 mg/kg + PA1647 25 mg/kg. In fact, such a combination resulted in a significantly higher reduction of 73% (line 4). So, the results are consistent with an additive or synergistic effect against schistosomules.

Then, due to this promising effect of PZQ and PA1647 on schistosomules, the PZQ/PA1647 association should be considered as a drug-association candidate for future clinical tests, after additional optimization on the association drug ratio, in order to target all parasite stages.

### 4. Microscopy

Adult *S. mansoni* females were treated in *vitro* with PA1259, praziquantel, artemether or mefloquine were examined by photon- and electron microscopy (SEM and TEM), with special focus on hemozoin, vitelline cells, musculature and tegument. In all cases, morphological alterations were apparent, but in highly variable extent and nature. The main observations reported in the Result Section were summarized in Table 3 where the most specific results have been indicated in bold. The following points are worth being emphasized.

**Table 3:** Relative intensity of damages on cultured *S. mansoni*, observed by electron microscopy.

	Treatment	PZQ	PA1259	MFQ	ARTM
Target	Hemozoin	++	**++++**	++	+
	Vitelline cells	+	++	**+++**	+
	Tegument	+	+++	+++	**++**
	Musculature	**+++**	+++	+++	-

#### a. Photon microscopy

Upon treatment by trioxaquine PA1259, adult females schistosomes readily regurgitated hemozoin, which is visible as a brown “cloud” in the right part of the Figure 3A. Few minutes after treatment, the remaining Hz, which was black in control worms (Figure 3D and 3E), turned to light brown color inside the gut of the treated worms (Figure 3B). Some time later, the gut walls of treated worms were no more visible, and the brown pigment invaded the whole worm body (Figure 3C). Such a modification was observed with none of the reference drugs, namely PZQ, ARTM, or MFQ. It strongly suggests that hemozoin is one of the targets of PA1259.

#### b. Scanning electron microscopy (SEM)

The tegument of trematodes is usually considered for its key role in nutrient absorption [33], secretory functions [34], and parasite protection against the host immune system [35]. Tegument is considered as the target (or one of the targets) of PZQ because it was shown by SEM that the biologically active levo-PZQ enantiomer was more prone to induce tegumental damages in *S. mansoni* than dextro-PZQ [36].

Worms treated with PA1259 were convoluted (Figures 3C and S1c). In fact, during the antischistosomal evaluation of trioxaquines, we have tested 90 trioxaquines (partial results have been reported in reference 12). With all active drugs of this series, the female *S. mansoni* worms adopted the shape of a loosen knot. It was so general, that it was possible to use this fact to detect trioxaquine-treated worms in blind assays [Boissier and Portela, unpublished work].

The disorganization of spines and the dilated spine sockets induced by PA1259 (Figure S2c and S3c, respectively) might be due to damages of the subjacent musculature of these retractile structures.

It should be noted that tegumental erosion and peeling upon *in vitro* treatment of *S. mekongi* [37] or *in vivo* treatment of other trematode parasite, such as *Clonorchis sinensis* [38], or *Fasciola hepatica* [39] by ARTM have been reported in the recent years.

Severe deformations of the tegument surface were seen in many worms, resulting in significant changes of the overall morphology of the worms. These facts suggest a drastic effect of drugs not only on the external tegument matrix, but also on the subjacent structures such as worm musculature. The observation of damages on a given part of the worms, namely hemozoin, tegument or suckers does not indicate that such a part of the worm is the location of the main target of the drug, even less the only drug target. Indirect widespread damages are indeed expected upon treatment in lethal conditions. However, we observed a significant number of differences between the damages induced by PZQ, ARTM, MFQ and PA1259, which are probably related to different mechanisms of action for these different drugs.

#### c. Transmission electron microscopy (TEM)

Beyond the general morphology and aspect of *S. mansoni* external structures, we examined the effect of the different drugs under a transmission electron microscope of ultrathin transverse sections of schistosomes. If the SEM study of trematode parasites has been well documented in literature, transmission-electron microscopy (TEM) studies are less common [40], [41].

Upon treatment with PA1259, MFQ or PZQ, the hemozoin content was significantly reduced with respect to control worms (Figure 6). A semi-quantitative estimation was carried out by counting the number of hemozoin pellets on a total surface of 200–300 µm^2^ in the mid-body region. The mean number of hemozoin pellets per µm^2^ was 1.6 for control schistosomes, 1.2 after treatment with ARTM, 0.8 and 0.6 after treatment with MFQ and PZQ, respectively, and close to 0.1 after treatment with PA1259. Then the gut hemozoin content of schistosomes treated by PA1259 was about 10% of that of untreated schistosomes. In addition, upon treatment by PA1259, a part of remaining hemozoin pellets was found lining the external epithelium membrane (Figure 6, panels e and f), suggesting perforation of the gut epithelium. However, the total hemozoin content in the gut lumen and epithelium was 0.2 pellet per µm^2^ after treatment with PA1259.

After treatment with PZQ, the tegumental matrix was slightly modified, but significant swelling of the underlying muscle bundles occurred (Figure 7, panels b). This feature argues that a main PZQ target should be musculature, instead of tegument, as previously reported [42]. TEM micrographs of worms treated by PA1259 exhibited complete disappearance of the tegument and severe swelling and disorganization of the sub-tegumental muscle (Figure 7, panels c). These images should be linked to SEM images, where worms became swollen, with retraction of spines into spine sockets, and extensive erosion of the tegument (Figure S3c and S5, respectively).

MFQ and PA1259 were the two drugs inducing the most severe damages to the tegument. These modifications may lead to exposure of worm surface antigen, resulting in attack of the parasite by host immune system. This fact had to be confirmed by investigation of the molecular processes responsible for the morphologic damages.

In previous reports, decrease of hemozoin gut content, as well as gut epithelium alteration, was observed in *S. mansoni* worms treated with quinine [43]. However, the authors did not observed alteration of tegument after quinine treatment. Extensive tegumental damage has been observed in both male and female worms treated with MFQ [29], [44]. Similar observations were obtained with *S. japonicum* parasite treated with MFQ [45]. Almost the same observations, (i) damage of tegumental and subtegumental structures, (ii) alteration of gut epithelial cells and (iii) fusion of vitelline balls were obtained in *S. mansoni* worms treated with artemether [46], but in a lower extent. Despite some common effects of drugs targeting heme, whatever their structural origins, trioxane-based, aminoquinoline-based or hybrid molecules (PA1259), on schistosomes, it is clear that the intensity of the effect and the morphological aspect after treatment vary according to the drug itself.

Upon treatment with PZQ, the most important damages were observed on the musculature of schistosomes, whereas tegument and vitelline cells were much less affected. MFQ was the most destructive drug, especially on vitelline cells. Upon treatment with PA1259, the more specific effect was a drastic decrease in hemozoin content, along with damages on the tegument and vitelline cells. The damages induced by artemether were much less severe and focalized on the tegument surface. This suggests certain specificity in the mode of action of each compound. The hybrid structure of trioxaquine PA1259 should explain interaction with heme polymerization, due to the 1,2,4-trioxane ring, and a severe effect on the musculature and tegument, as shown with the quinolone-based MFQ.

### 5. Molecular mechanism of damages induced by antischistosomal drugs

#### a. Reduced or radical oxygen species: NO•, O_2_•^−^, H_2_O_2_

Quantification of ROS was performed on adult worms at a drug concentration giving the same proportion of immobilized worms, for PZQ and PA1259 [28a]. Results are reported in Figure 8. In worms treated with PZQ, the production of O_2_•^−^ was twice higher than that of control worms. By contrast, treatment with PA1259 did not improve the production of O_2_•^−^ with respect to control (Figure 8A). The production of H_2_O_2_ production whether control, PZQ, or PA1259 was similar (Figure 8B). The most significant feature was NO• production, which was very low in control worms. Females treated with PZQ exhibited a slightly enhanced production of NO• (×1.5) near the tegument, whereas, in females treated with PA1259, the fluorescence due to NO• was very high (×4.3 with respect to control worms) and mainly detected inside the gut and near the vitelline cells (Figure 8C). Then, the reactive oxygen species produced are different, in nature, in localization, and in quantities, when worms are treated with either PZQ or PA1259. These results strongly suggest that these drugs have different targets and mechanisms of action on schistosomes. Oxadiazoles may also act as nitric oxide donors [6b]. Quantification of gene transcripts showed that anti-oxidant metabolism is increased when worms are exposed to a sub-lethal dose of PZQ. Our results are consistent with reference 47.

#### b. Characterization of covalent heme-drug adducts

The alkylation ability of the drug toward heme was investigated in *S. mansoni* treated with ARTM or PA1259. In both cases, heme-drug adducts were detected by LC-MS analysis of the worm extracts.

**Figure 11:**
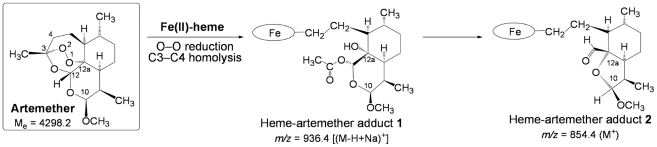
Mechanism of alkylation of heme by artemether (the oval stands for the protoporphyrin-IX ligand).

In the extract of worms treated by ARTM (Figure 9), the product having ionic current *m/z* = 854.4 amu is consistent with the formation of heme-artemether covalent adducts **2** depicted in Figure 11. The reductive activation of the O–O bond of artemether by iron(II)-heme, followed by homolytic cleavage of the C3–C4 bond, resulted in the formation of a primary alkyl radical centered at C4. As previously reported with the heme model manganese(II)-tetraphenylporphyrin, this radical was able to alkylate the porphyrin macrocycle, leading to covalent heme-drug adducts [48]. As for artemisinin, alkylation of heme by the drug can indeed occur on the four *meso* positions of the porphyrin macrocycle, giving rise to regioisomeric adducts **1**. The “South” cycle of the artemether moiety was subsequently rearranged by nucleophilic attack of the hydroxyl function at C12a onto the acetal C10, to generate the heme-artemether adduct **2**, bearing a 5-membered lactone. Such a mechanism is very similar to the rearrangement reported for artemisinin derivatives having an amine function at C10 [49]. Trace amounts of “complete” covalent heme-artemether adducts **1** (M_e_ = 914.4 amu), were also detected, with *m/z* = 936.4 amu, corresponding to the substitution of a proton of a propionic side chain of heme by sodium [(M-H+Na)^+^].

**Figure 12:**
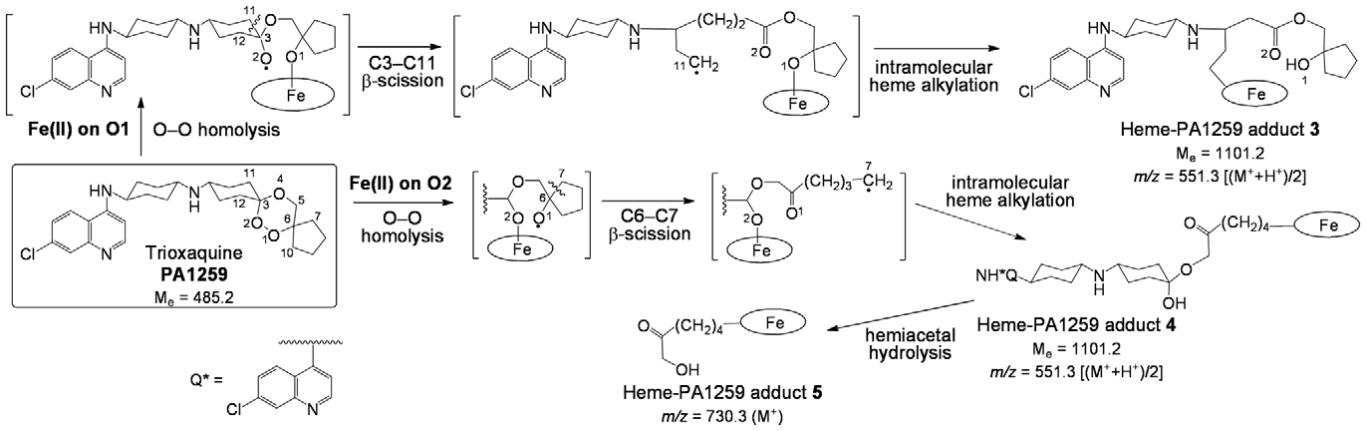
Mechanism of alkylation of heme by trioxaquine PA1259 (the oval stands for the protoporphyrin-IX ligand).

The LC-MS analyses of extracts of *S. mansoni* worms treated with PA1259 are reported in Figure 10a–c. The exact mass value of 1101.2 amu (Figure 10b) corresponds to the mass of heme (616.2) plus the mass of PA1259 (485.2), and can be assigned to covalent adducts between heme and PA1259. The structures and mechanisms of formation of these adducts are depicted in Figure 12. It should be noted that the inner-sphere reductive activation of the peroxide bond of PA1259 can occur with coordination of iron(II)-heme either on O1 or O2, giving rise to the formation of alkoxy radicals either on O2 or O1, respectively. Subsequent β-scission of the adjacent C3–C11 or C6–C7 bond, respectively, generated C-centered radicals able to alkylate heme and provide the covalent heme-drug adducts **3** and **4**. The covalent adduct **5**, resulting from the hydrolysis of the hemiacetal function of adduct **4**, was also detected (Figure 10c). In addition, the isotopic patterns of signals at *m/z* 551.3 and 730.3 clearly showed that the corresponding adducts contained one iron atom (M-1 at 550.3 and M-2 at 728.2, respectively, due to ^54^Fe). By contrast, one chlorine atom was detected in adducts at *m/z* 551.3 (M+1 at 552.3 due to ^37^Cl), whereas adduct **5** contained no chlorine after release of the 7′-chloro-4′-aminoquinoline moiety (Figure 12). The heme-drug adducts **2–5** were undetectable in all extracts of untreated *S. mansoni* worms. Because the worms treated with ARTM or PA1259 were carefully washed before lyophilization, the detected adducts were clearly contained inside the worms, and cannot be considered as an external contamination.

#### c. Heme oxidative cleavage in *S. mansoni*

**Figure 13:**
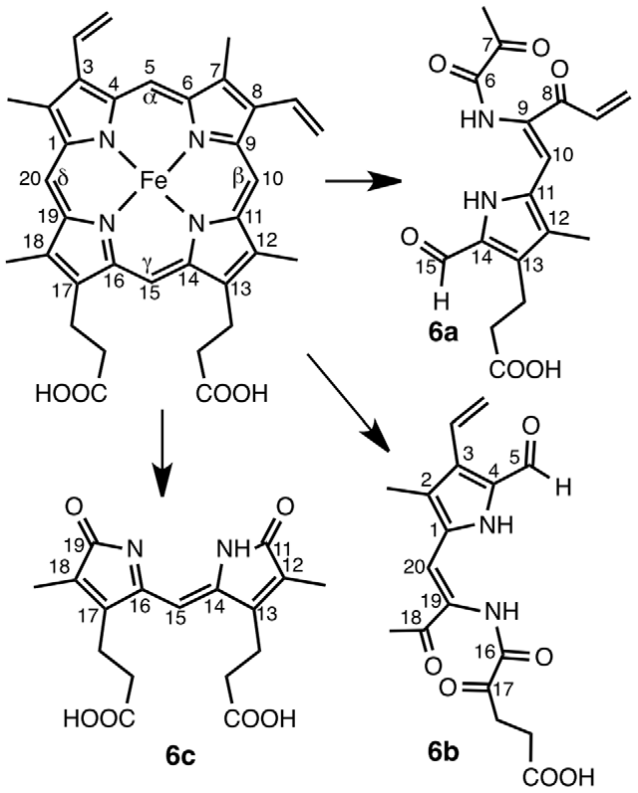
Oxidative cleavage of heme to fragment with *m/z* = 347.2 (M^+^).

A heme oxidase activity has been previously reported in *S. japonicum* [50], which may account to supply iron to schistosomes by catabolism of the heme of the host. Then, we were interested to detect heme fragments that might result from the possible oxidative cleavage of the heme macrocycle by the parasites. The LC-MS analysis of untreated worms showed the presence of two chromatographic peaks with the same *m/z* value of 347.1 amu. These peaks were also produced (with same mass spectrum and retention time values) by reaction of hemin or β-hematin, the synthetic analogue of hemozoin, in the presence of hydrogen peroxide [El Rez, Pradines and Robert, unpublished data]. The mass and UV absorption (*λ*_m_ = 280 nm) of these heme fragments suggest a common dipyrrolic structure. Possible structures are depicted in Figure 13, which result from oxidative cleavage of C5–C6, C7–C8, and C15–C16 (**6a**), C5–C6, C15–C16, and C17–C18 (**6b**), or C10–C11 and C19–C20 (**6c**) of heme ligand, in a similar way as the cleavage protoporphyrin-IX to biliverdin-IX by mammalian heme oxygenases. Obviously, the cleavage of the heme ring results in a drastic loss of visible absorbance, and discoloration of the pigment. Interestingly, treatment with ARTM or MFQ did not produce a significant amount of that heme fragment.

### Conclusion

A multidisciplinary approach allowed us to propose the following mode of action of the antischistosomal trioxaquine PA1259. First, the easy absorption due to the quinoline moiety of PA1259 should allow the drug to interact with the schistosome process of polymerization of heme to hemozoin. The reaction of the peroxide function of trioxaquine with free heme resulting from the degradation of hemoglobin was assessed by the production of heme-drug adducts characterized in worm extracts. As heme-drug adducts are probably able to inhibit the heme polymerization, and are themselves unable to polymerize (this feature has been demonstrated for heme-artemisinin adducts [51]), this reaction led to accumulation in the worm of soluble redox-active heme derivatives. Then, the iron chelated by the protoporphyrin-IX ligand of heme or heme-drug adducts induced the production of reactive oxygen species, which should readily destroy hemozoin by radical chain reactions, resulting in (i) drastic discoloration of worms from black to brown, and (ii) much lower density of hemozoin in worms treated with PA1259 than in control worms, as observed in microscopy (Figures 3 and 6).

The high quantity of nitric oxide detected in the parasite gut is certainly an important element of this oxidative stress (Figure 8). Third, this oxidative stress would cause gastrodermis perforation and the passage of the gut content through the gastrodermis, as observed by electronic microscopy (Figure 6). The oxidative cleavage of heme also releases free iron that should increase the production of radical oxygen species. Finally, the substructure of the tegument was altered by the invasion of oxygen reactive species (Figures 4 and 7).

However, several questions remain to be adressed. Indeed, the fact that cercariae are heavily affected by treatment, the external tegumental alteration, and the effect on vitelline cells observed with PA1259 are not directly explained by reaction of the drug with heme. But the sequence hemoglobin/heme/hemozoin is not the single source of iron in schistosomes and several other iron proteins may play a role. Schistosomes have high demand for iron and are dependent on host iron for early development within the mammalian host [52]. In each stage, from miracidia to adult, schistosomes express tegumental divalent metal transporters involved in iron uptake [53]. Moreover, schistosomes are known to bind host transferrin at their surface [52]. The parasite also possesses two ferritin isoforms involved in storage and release of iron [54], [55]. One isoform, called yolk-ferritin or ferritin 1, is predominantly found in mature egg laying female, where it has been localized in the vitelline cells and in the ovary [56]. Vitelline stores of iron are implicated in eggshell formation [57]. Interestingly, the two isoforms of ferritin are also expressed in the parasite egg suggesting these molecules are also synthesized by embryo [57]. These observations emphasize the importance of iron in schistosome metabolism and egg formation, even on worm stages that do not ingest hemoglobin yet. Ferritin has not yet been characterized in cercariae, however the expressed sequence tags of ferritin 2 are available in sequence database suggesting that cercariae express this enzyme. Several recent articles suggest that iron metabolism should be a valuable target for either chemotherapy or vaccine development against schistosomes [58], [59].

Using an anti-schistosomal drug that is also active on malaria parasites is a real matter of discussion [PA1259 is curative on *P. vinckei petteri* infected mice orally treated at 25 mg/kg/day during four days (5/5 mice cured without recrudescence at Day-30, unpublished data from Palumed)]. Such dual activity might be considered as an advantage since patients are suffering from both diseases in many endemic areas. One the other side, one can argue about the possibility to generate trioxane-resistant strains of *P. falciparum*. But we should keep in mind that no trioxaquine-resistant strain has been selected after two years of drug pressure (unpublished results).

In conclusion, trioxaquine PA1259 is the most active against schistosomes among the trioxane-containing drugs that have been tested up to now. Its phosphate salt, PA1647 acts in synergy with praziquantel, specially against schistosomules, when infected mice are treated by oral administration. This opens the route to an efficient bitherapy of a highly neglected disease.

## Supporting Information

Figure S1 **SEM images of** ***S. mansoni*** **adult females.** Control worms (a), compared to worms treated with b) praziquantel (PZQ), c) trioxaquine PA1259, d) mefloquine (MFQ), or e) artemether (ARTM). White crosses are breaks related to the preparation for microscopy.
(TIF)

Figure S2 **SEM images of the dorsal face of the head region of** ***S. mansoni*** **adult females.** Control worms (a), compared to worms treated with b) praziquantel (PZQ), c) trioxaquine PA1259, d) mefloquine (MFQ), or e) artemether (ARTM). Magnification ×5000; the bars stand for 1 µm. Sensory papillae are noted sp. Crystals caused by buffer are noted buf.
(TIF)

Figure S3 **SEM images of the mid-body region of** ***S. mansoni*** **adult females.** Control worms (a), compared to worms treated with b) praziquantel (PZQ), c) trioxaquine PA1259, d) mefloquine (MFQ), or e) artemether (ARTM). Magnification ×5000; the scale bars stand for 1 µm.
(TIF)

Figure S4 **SEM images of the mid-body region of** ***S. mansoni*** **adult females.** Control worms (a), compared to worms treated with b) praziquantel (PZQ), c) trioxaquine PA1259, d) mefloquine (MFQ), or e) artemether (ARTM). Magnification ×1000; the scale bars stand for 10 µm.
(TIF)

Figure S5 **SEM images of tegumental damages in** ***S. mansoni*** **adult females.** Worms treated with a) trioxaquine PA1259, or b) artemether (ARTM) (tail region). Magnification ×2000 (a1,b1) or ×5000 (a2,b2); the scale bars stand for 10 µm (a1-b1), or for 1 µm (a2-b2).
(TIF)

Figure S6 **TEM images of vitelline cells (vc) containing vitelline balls (vb).** Control worms (a), compared to worms treated with b) praziquantel (PZQ), c) trioxaquine PA1259, d) mefloquine (MFQ), or e) artemether (ARTM). The scale bars stand for 1 µm in panels a1-e1, and 2 µm in panels a2-e2. Fusion of vitelline balls (vb) in vitelline droplets (vd); ld stands for lipid droplets.
(TIF)

Figure S7 **SEM images of the tail region of** ***S. mansoni*** **adult females.** Control worms (a), compared to worms treated with b) trioxaquine PA1259. Magnification ×2000 (a1, b1) or ×5000 (a2, b2). The scale bars stand for 10 µm (a1, b1) or 1 µm (a2, b2).
(TIF)
