# Supplementary figures and images for: Antischistosomal Activity of Trioxaquines: In Vivo Efficacy and Mechanism of Action on Schistosoma mansoni
Source: PLoS Negl Trop Dis. 2012 Feb 14;6(2):e1474. doi: 10.1371/journal.pntd.0001474 (PMC3279339; doi:10.1371/journal.pntd.0001474)

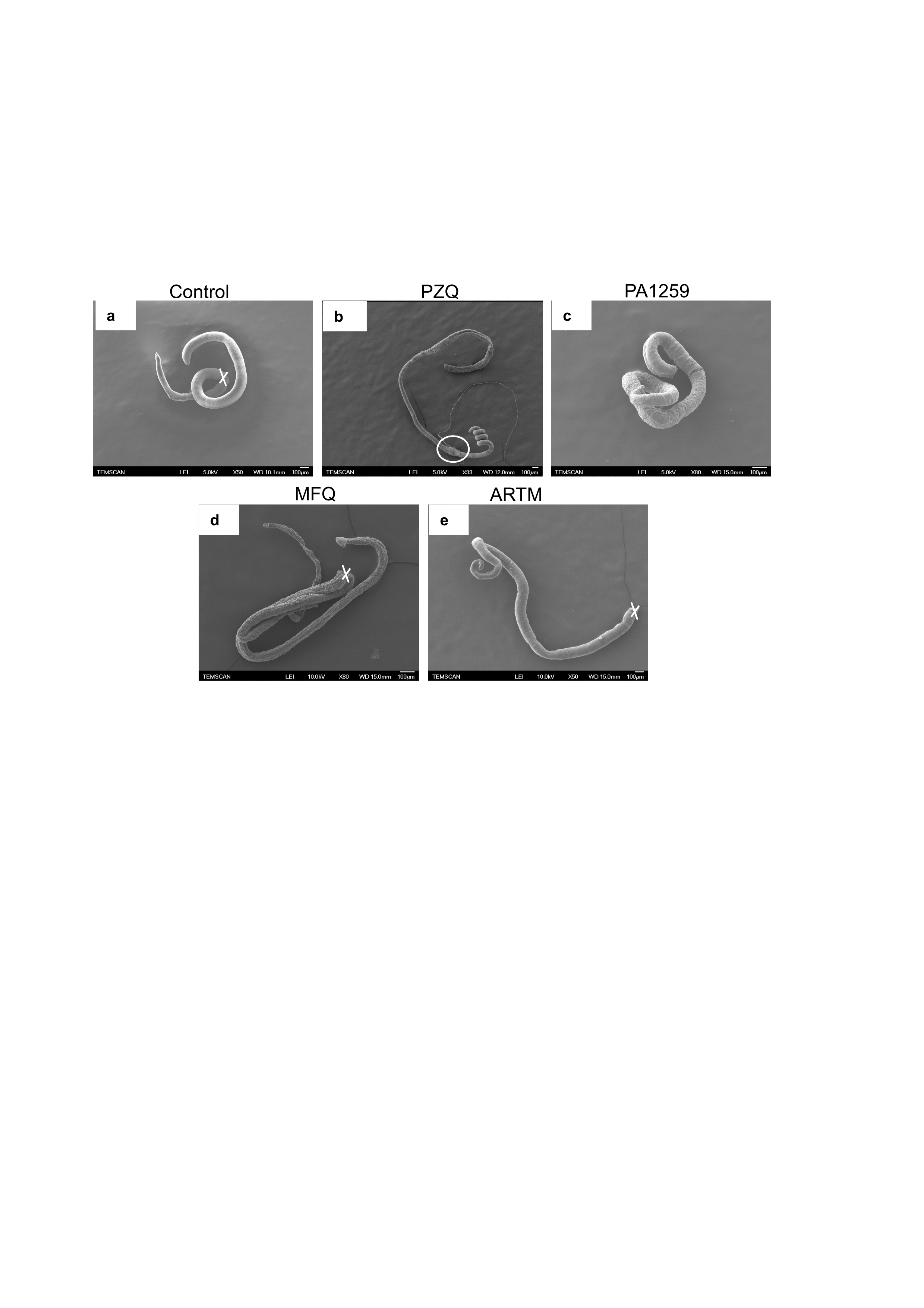

Supplement: Figure S1 — SEM images of S. mansoni adult females. Control worms (a), compared to worms treated with b) praziquantel (PZQ), c) trioxaquine PA1259, d) mefloquine (MFQ), or e) artemether (ARTM). White crosses are breaks related to the preparation for microscopy. (TIF) [file pntd.0001474.s001.tif]

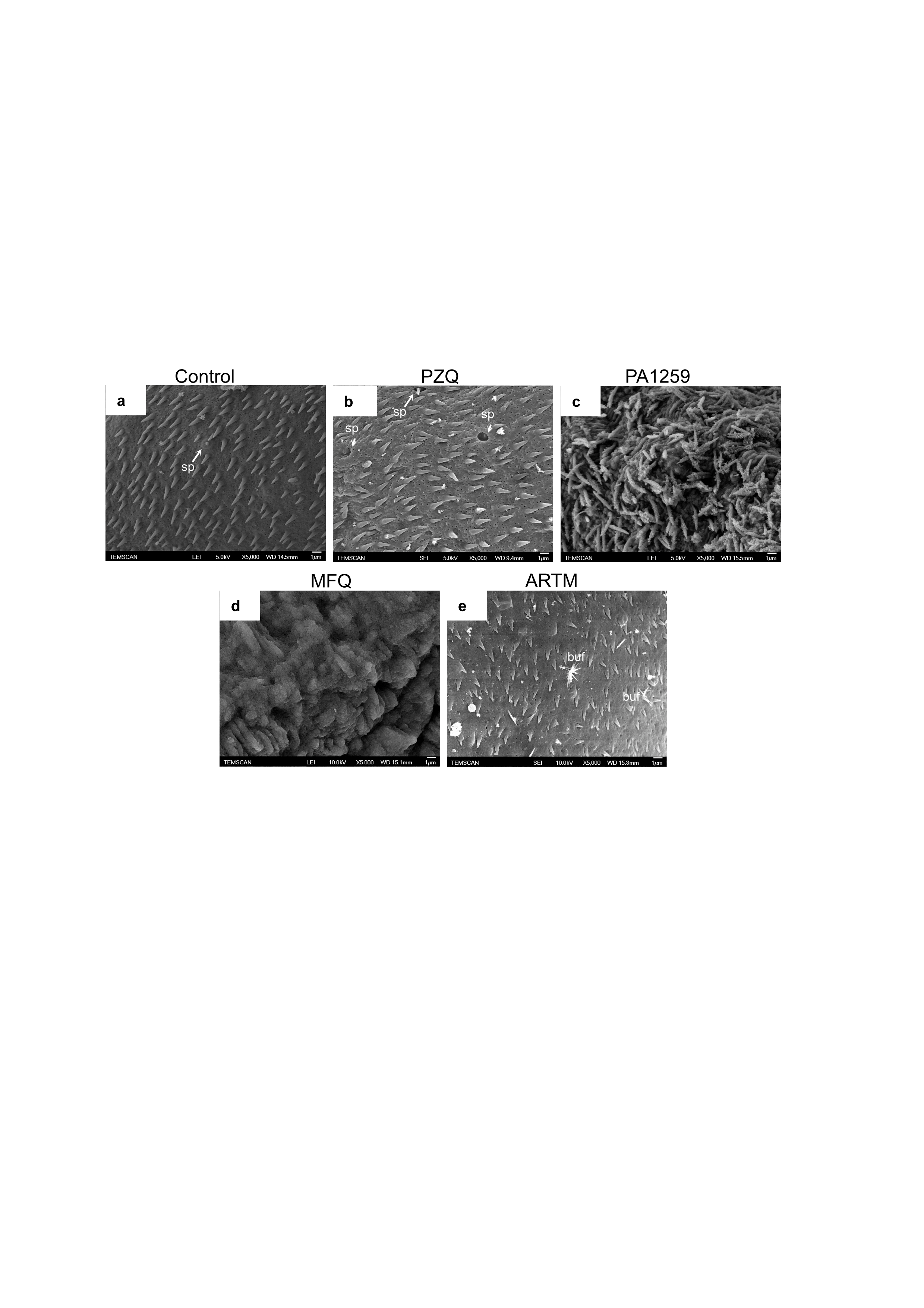

Supplement: Figure S2 — SEM images of the dorsal face of the head region of S. mansoni adult females. Control worms (a), compared to worms treated with b) praziquantel (PZQ), c) trioxaquine PA1259, d) mefloquine (MFQ), or e) artemether (ARTM). Magnification ×5000; the bars stand for 1 µm. Sensory papillae are noted sp. Crystals caused by buffer are noted buf. (TIF) [file pntd.0001474.s002.tif]

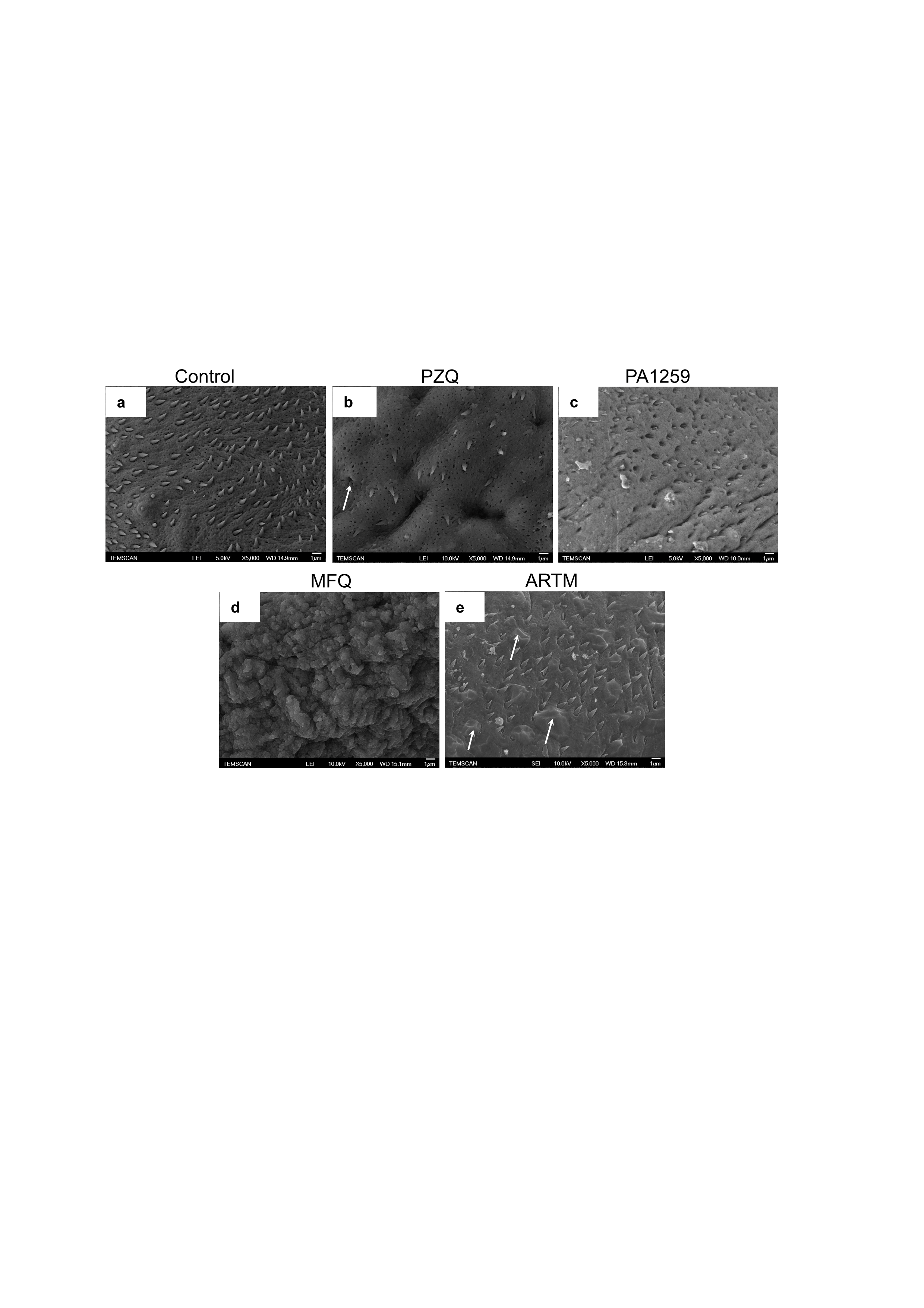

Supplement: Figure S3 — SEM images of the mid-body region of S. mansoni adult females. Control worms (a), compared to worms treated with b) praziquantel (PZQ), c) trioxaquine PA1259, d) mefloquine (MFQ), or e) artemether (ARTM). Magnification ×5000; the scale bars stand for 1 µm. (TIF) [file pntd.0001474.s003.tif]

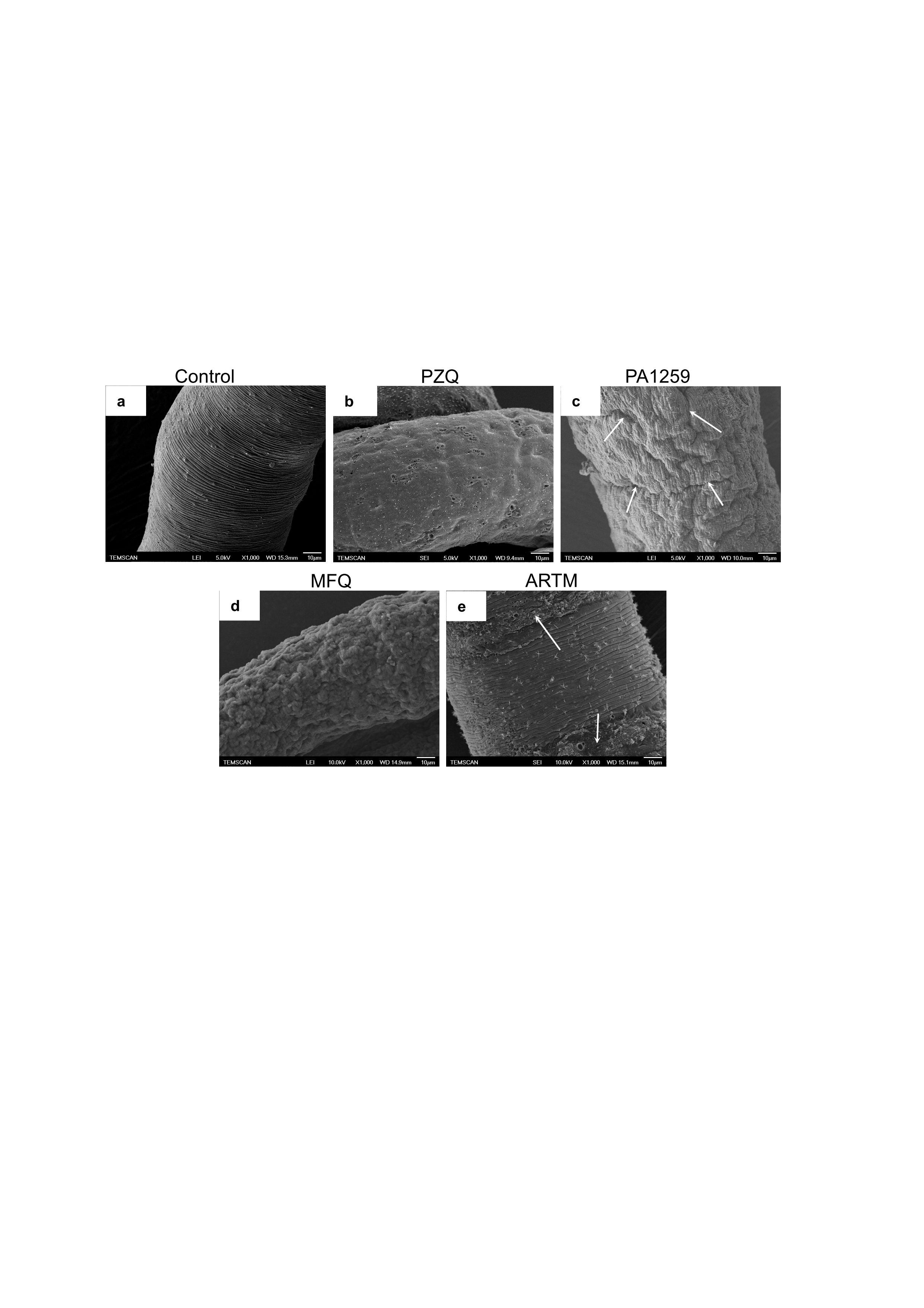

Supplement: Figure S4 — SEM images of the mid-body region of S. mansoni adult females. Control worms (a), compared to worms treated with b) praziquantel (PZQ), c) trioxaquine PA1259, d) mefloquine (MFQ), or e) artemether (ARTM). Magnification ×1000; the scale bars stand for 10 µm. (TIF) [file pntd.0001474.s004.tif]

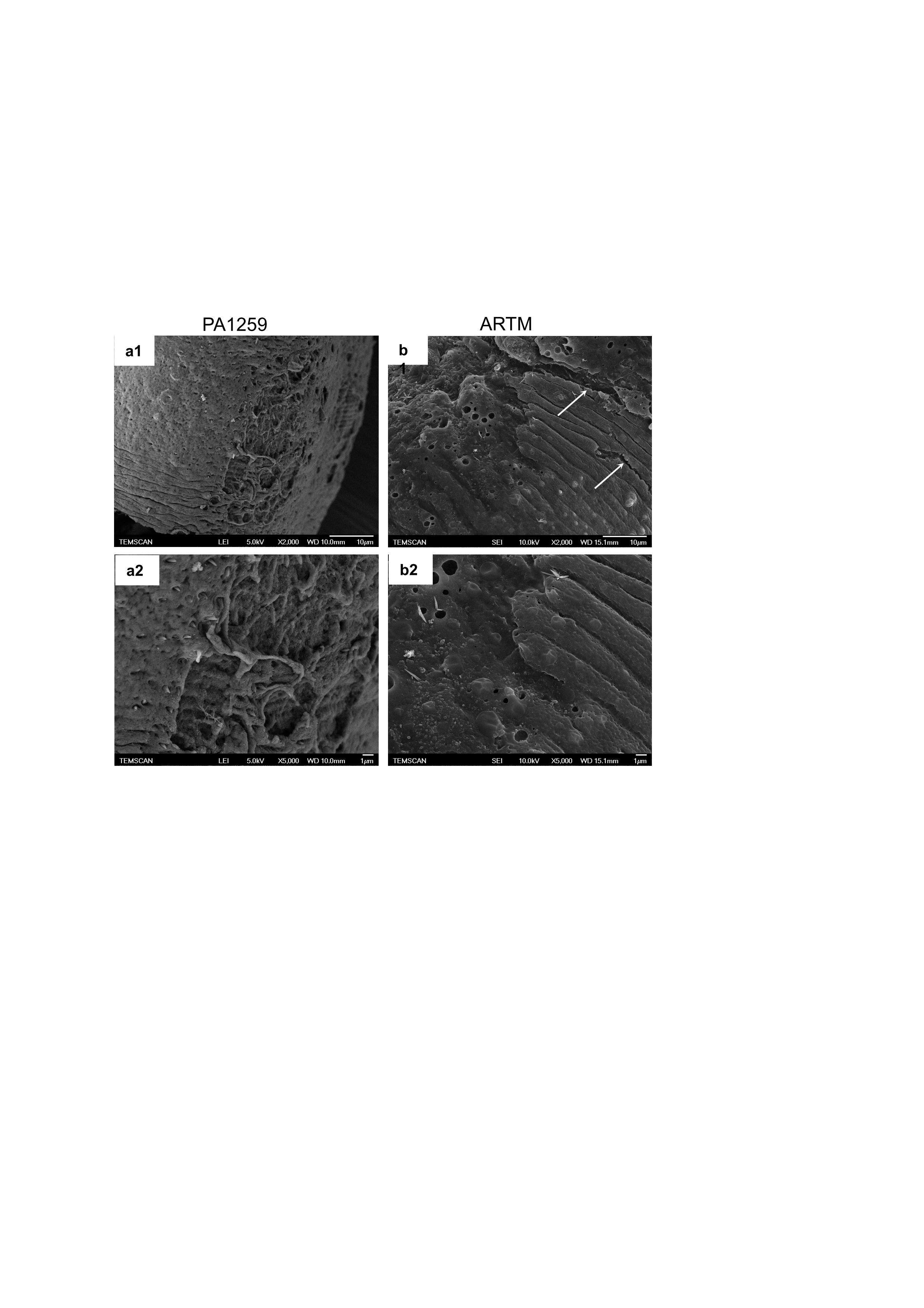

Supplement: Figure S5 — SEM images of tegumental damages in S. mansoni adult females. Worms treated with a) trioxaquine PA1259, or b) artemether (ARTM) (tail region). Magnification ×2000 (a1,b1) or ×5000 (a2,b2); the scale bars stand for 10 µm (a1-b1), or for 1 µm (a2-b2). (TIF) [file pntd.0001474.s005.tif]

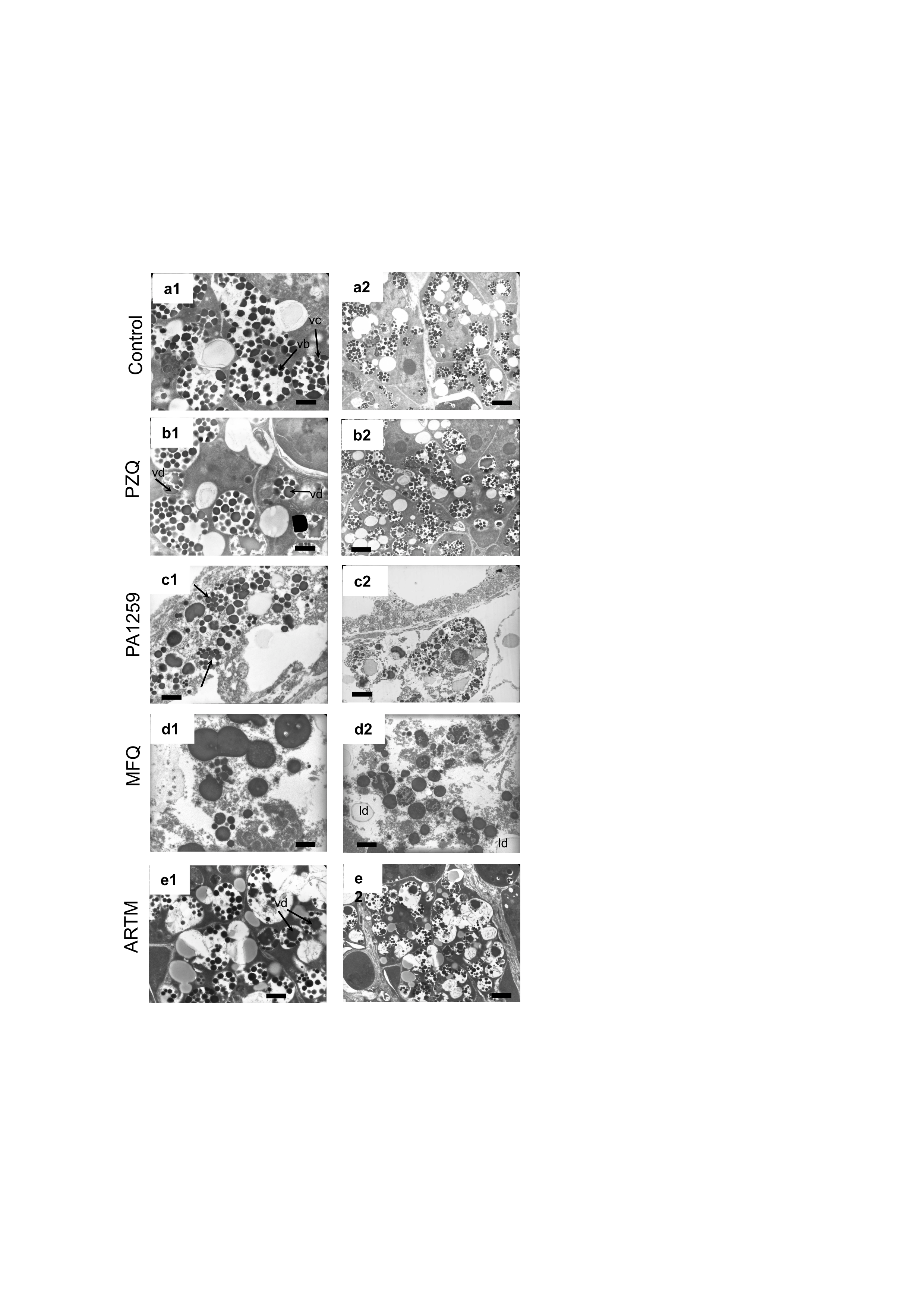

Supplement: Figure S6 — TEM images of vitelline cells (vc) containing vitelline balls (vb). Control worms (a), compared to worms treated with b) praziquantel (PZQ), c) trioxaquine PA1259, d) mefloquine (MFQ), or e) artemether (ARTM). The scale bars stand for 1 µm in panels a1-e1, and 2 µm in panels a2-e2. Fusion of vitelline balls (vb) in vitelline droplets (vd); ld stands for lipid droplets. (TIF) [file pntd.0001474.s006.tif]

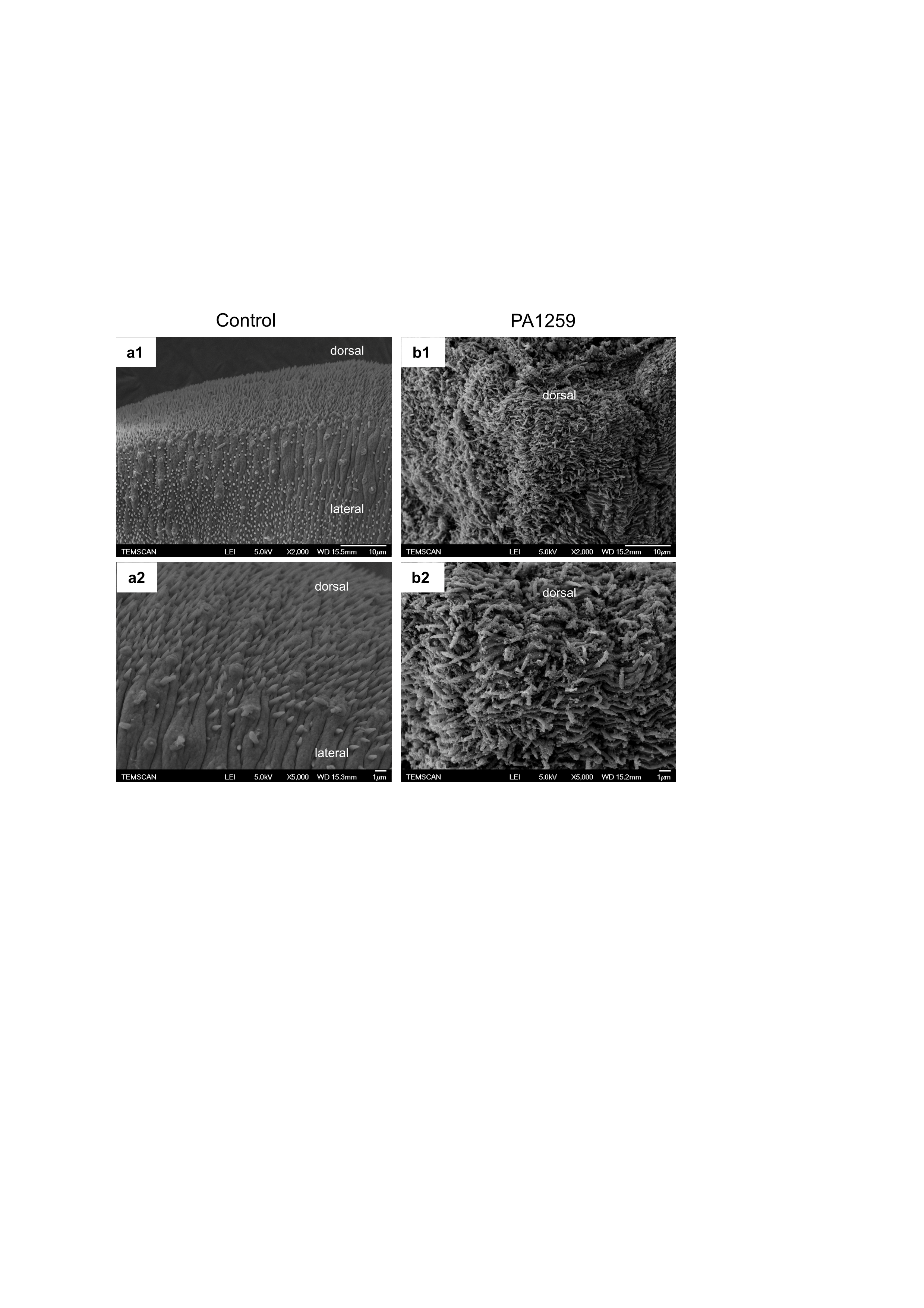

Supplement: Figure S7 — SEM images of the tail region of S. mansoni adult females. Control worms (a), compared to worms treated with b) trioxaquine PA1259. Magnification ×2000 (a1, b1) or ×5000 (a2, b2). The scale bars stand for 10 µm (a1, b1) or 1 µm (a2, b2). (TIF) [file pntd.0001474.s007.tif]
